# Role of miR‐466 in mesenchymal stromal cell derived extracellular vesicles treating inoculation pneumonia caused by multidrug‐resistant *Pseudomonas aeruginosa*

**DOI:** 10.1002/ctm2.287

**Published:** 2021-01-13

**Authors:** Meng‐meng Shi, Ying‐gang Zhu, Jia‐yang Yan, Jean‐Jacques Rouby, Hanssa Summah, Antoine Monsel, Jie‐ming Qu

**Affiliations:** ^1^ Department of Respiratory and Critical Care Medicine, Ruijin Hospital, School of Medicine Shanghai Jiao Tong University Shanghai China; ^2^ Institute of Respiratory Diseases, School of Medicine Shanghai Jiao Tong University Shanghai China; ^3^ Department of Pulmonary and Critical Care Medicine, Hua‐dong Hospital Fudan University Shanghai China; ^4^ Multidisciplinary Intensive Care Unit, Department of Anesthesiology and Critical Care, La Pitié‐Salpêtrière Hospital, Assistance Publique‐Hôpitaux de Paris (APHP) Sorbonne University Paris France; ^5^ INSERM, UMR S 959, Immunology‐Immunopathology‐ Immunotherapy (I3) Sorbonne Université Paris F‐75005 France; ^6^ Biotherapy (CIC‐BTi) and Inflammation‐Immunopathology‐Biotherapy Department (DHU i2B) Hôpital Pitié‐Salpêtrière AP‐HP Paris F‐75651 France

**Keywords:** extracellular vesicles, mesenchymal stromal cell, micoRNA, multidrug‐resistant pseudomonas aeruginosa, pneumonia

## Abstract

**Rationale:**

The effects of mesenchymal stromal cells (MSCs) and MSC‐derived extracellular vesicles (MSC EVs) on multidrug‐resistant *pseudomonas aeruginosa* (MDR‐PA)‐induced pneumonia remain unclear.

**Materials and methods:**

MicroRNA array and RT‐PCR were used to select the major microRNA in MSC EVs. Human peripheral blood monocytes were obtained and isolated from qualified patients. The crosstalk between MSCs/MSC EVs and macrophages in vitro was studied. MDR‐PA pneumonia models were further established in C57BL/6 mice and MSC EVs or miR‐466 overexpressing MSC EVs were intratracheally instilled.

**Results:**

MiR‐466 was highly expressed in MSC EVs. MSCs and miR‐466 promoted macrophage polarization toward Type 2 phenotype through TIRAP‐MyD88‐NFκB axis. Moreover, cocultured macrophages with miR‐466 overexpressing MSCs significantly increased the phagocytosis of macrophages. MSC EVs significantly reduced mortality and decreased influx of BALF neutrophils, proinflammatory factor levels, protein, and bacterial load in murine MDR‐PA pneumonia. Administration of miR‐466 overexpressing MSC EVs further alleviated the inflammatory severity.

**Conclusions:**

MSC‐derived EVs containing high levels of miR‐466 may partly participate in host immune responses to MDR‐PA. Both MSCs and MSC EVs have therapeutic effects in treating MDR‐PA‐induced pneumonia.

## Graphical abstract

Ad‐MSC derived EVs contain high level of miR‐466. Both Ad‐MSCs and Ad‐MSC EVs have therapeutic effects in treating MDR‐PA‐induced pneumonia. miR‐466 promotes phenotype switch to M2 type of macrophages, exerting immunomodulatory effects.

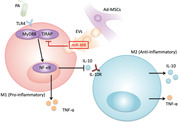

## 1 INTRODUCTION

Hospital‐acquired pneumonia and ventilator‐associated pneumonia generate a significant burden in terms of mortality, morbidity, and hospital costs.^1^, ^2^ Notably, multidrug‐resistant (MDR) bacteria pneumonia can lead to septic shock, acute respiratory distress syndrome, and death.^3^ *Pseudomonas aeruginosa* (PA), a Gram‐negative rod whose resistance to cephalosporins and carbapenems is a growing pervasive problem, causes hospital‐acquired and ventilator‐associated pneumonia in 16–20% of critically ill patients.^4^ A nationwide study reported that multidrug‐resistant strains account for 23% of all PA isolates from 2008 to 2010 in China.^5^ This antibiotic resistance to PA has resulted in a clinically significant impact on key Intensive Care Unit (ICU) outcomes, including length of hospital stay, cost, and mortality.^3^ Development of new antipseudomonal antibiotics is limited and new strategies for treating multidrug‐resistant PA (MDR‐PA) are needed.

Many experimental studies demonstrated that mesenchymal stromal cells (MSCs) and their derived extracellular vesicles (EVs) significantly reduce lung inflammation resulting from different types of noninfectious lung injury^6^, ^7^, ^8^, ^9^, ^10^ allowing phase I/II trials in humans.^11^, ^12^ There is also a large body of evidence demonstrating that lung inflammation resulting from tracheal inoculation of live antibiotic sensitive *Escherichia coli* ^13^, ^14^, ^16^, ^17^, ^18^, ^19^ and PA^15^ is significantly reduced by MSCs^13^, ^14^, ^15^, ^16^, ^17^, ^18^, ^19^ and MSCs‐derived EVs.^20^, ^21^ In addition, macrophage phagocytosis,^14^, ^15^, ^16^, ^17^, ^20^, ^21^ bacterial killing,^13^, ^14^, ^15^, ^16^, ^17^, ^18^, ^19^, ^20^, ^21^ and outcome^13^, ^15^, ^16^, ^18^, ^19^, ^20^ are improved. It is highly likely that MSC‐EVs have the same therapeutic effect on inoculation pneumonia caused by multidrug‐resistant bacteria. To confirm such a benefit that could open new therapeutic options in critically ill patients with ventilator‐associated pneumonia caused by MDR‐PA, we decided to evaluate the efficacy of MSC‐EVs in a murine model of MDR‐PA pneumonia. As in *E. coli* endotoxin induced acute lung injury, the anti‐inflammatory effect of human‐MSC EVs was only partially explained by the transfer of keratinocyte growth factor (KGF) and angiopoietin‐1 (Ang‐1) mRNA into injured cells,^9^, ^22^ we hypothesized that other transferable genetic materials such as microRNAs^23^, ^24^ could be involved in the therapeutic benefit of MSC EVs in MDR‐PA inoculation pneumonia. This study was then designed to screen in vitro the expression of microRNAs within MSC EVs and subsequently determine in vivo whether the therapeutic effects of MSC EVs in MDR‐PA inoculation pneumonia were mediated by identified microRNAs.

## 2 MATERIALS AND METHODS

### 2.1 Study subjects and animals

#### 2.1.1 Primary cells and cell lines cultures

Mouse adipose‐derived MSCs (Ad‐MSCs) were isolated from mouse adipose tissue in line with previously reported methods.^15^ Human bone marrow mesenchymal stem cells (h‐MSCs) were obtained from ATCC. RAW264.7 mouse macrophage cells (Catalog #TCM13, http://www.cellbank.org.cn/detail_1.asp?id=217&serial=TCM13) and NCTC clone 929 cells (L cell, L‐929, derivative of Strain L) (Catalog #GNM28, http://www.cellbank.org.cn/detail_1.asp?id=352&serial=GNM28) were obtained from the cell bank of the Chinese Academy of Sciences. Human peripheral blood monocytes were isolated from human peripheral blood by using magnetic bead separation methods according to manufacturers’ protocol as described in the Supporting Information. We included six patients with MDR‐PA‐induced pneumonia and six healthy volunteers in Ruijin Hospital, School of Medicine, Shanghai Jiao Tong University. Their demographic information is showed in Table S3.

#### 2.1.2 Isolation and characterization of Ad‐MSC EVs

Ad‐MSC EVs were isolated from the supernatant of Ad‐MSCs and L‐929s as described before.^9^, ^22^ Ad‐MSCs or L‐929s were cultured until 80–90% confluent in P75 flasks and starved for 48 h in DMEM/F12 or RPMI1640 (Gibco, Thermo Fisher Scientific, Waltham, MA; http://www.thermofisher.com) without serum. To isolate EVs, the supernatant of Ad‐MSCs or L‐929s was first centrifuged at 1400 *g* for 20 min to remove cellular debris, then at 100 000 *g* (XPN‐100, Beckman Coulter, CA) to sediment the EVs for 1.5 h at 4°C, washed in phosphate‐buffered saline (PBS), and submitted to a repeated ultracentrifugation for another 1.5 h at 4°C. Ad‐MSCs or L‐929 EVs were resuspended according to the final count of Ad‐MSCs or L‐929s after 48 h of serum starvation (10 μl per 1 × 10^6^ cells) and stored at −80°C until further use. In addition, EVs derived from Ad‐MSCs transfected with either miR‐466 mimics (miR‐466‐m‐MSC EVs) or miR‐466 mimics negative control (NS‐m‐MSC EVs) were also collected following the same protocol as above. The size and concentration of EVs were determined by nanoparticle tracking analysis (NTA) (Shanghai XP Biomed Ltd., Shanghai, China). The EVs were identified by transmission electronic microscope analysis by Shanghai XP Biomed Ltd. EVs, isolated from 60 ml supernatant of Ad‐MSCs were fixed in 1.5 M sodium cacodylate buffer (pH 7.4), absorbed onto formvar‐coated copper grids (Science Services, München, Germany), and examined by an electronic microscope (TecnaiTM G2 Spirit; FEI Company, Hillsboro, OR). Images were taken with an AMT digital camera for data acquisition.

#### 2.1.3 Mice

Male C57BL/6 mice (8–10 weeks) were obtained from Charles River Labs, Beijing, China. They were fed in an SPF facility at the Research Center for Experimental Medicine of Ruijin Hospital, Shanghai Jiao Tong University School of Medicine, China. All animal procedures were approved by Ruijin Hospital Animal Ethics Committee.

#### 2.1.4 Identification and preparation of clinical strain

The MDR‐PA strain, collected from Ruijin Hospital, Shanghai Jiao Tong University School of Medicine, was isolated from the sputum of a patient with pneumonia. Antimicrobial susceptibility testing for this MDR‐PA strain was determined by e‐test, and the detailed antimicrobial resistance profile is presented in Figure S1. Genome analysis was also conducted by next‐generation sequencing (Shanghai Personal Biotechnology Co., Ltd. https://www.personalbio.cn) and the MDR‐PA strain was demonstrated to match with serotype of O11 (Accession number 104721.1) by BLAST (Basic Local Alignment Search Tool; https://blast.ncbi.nlm.nih.gov).^26^, ^27^ The PA transfected with Green Fluorescent Protein (GFP‐PA) was kindly provided by Prof. Song Yuan‐Lin (Zhongshan Hospital, Fudan University, Shanghai, China).

### 2.2 Methods

#### 2.2.1 Co‐culture with Ad‐MSCs and human peripheral blood monocytes or murine macrophages

Human peripheral blood monocytes or murine macrophages (5 × 10^5^ cells/well) were seeded in 12‐well plates with Ad‐MSCs (2.5 × 10^5^ cells/well) seeding in the upper compartment of Transwells (0.4 μm pore size, Costar, Corning, Sigma‐Aldrich, St. Louis, MO) or coincubated with Ad‐MSC EVs (25 μl Ad‐MSC EVs) for 24 h and then exposed to MDR‐PA with multiplicity of infection (MOI) (bacteria: cells) of 1. All the supernatants were subsequently collected for enzyme‐linked immunosorbent assay (ELISA) and the macrophages were collected for RNA extraction or flow cytometry analysis. In separate experiments, Ad‐MSCs were pretreated with the neutral sphingomyelinase (nSMase) inhibitor, GW4869 (10 μM) (Sigma‐Aldrich),^25^ known as the inhibitor of EV secretion, for 4 h and Ad‐MSC EVs were pretreated with anti‐CD44 (1 μg/ml) (BD Pharmingen, San Diego, CA), known as inhibitor of EV uptake, for 15 min at 4°C before coincubating with macrophages.

In order to explore the crosstalk between macrophages and Ad‐MSC EVs through endocytosis, Ad‐MSC EVs were pretreated with Triton‐X100 (Invitrogen, Thermo Fisher Scientific, Waltham, MA, which is well known as reagent of permeabilization, for 10 min at room temperature before incubating with macrophages. Macrophages were pretreated with chlorpromazine hydrochloride (10 μM) (Sigma‐Aldrich), which is well known as blocking endocytosis. The cells then incubated with Ad‐MSC EVs for 24 h and exposed to MDR‐PA with MOI (bacteria: cells) of 1. All the supernatants were subsequently collected for ELISA assay.

#### 2.2.2 Flow cytometry analysis

MDR‐PA‐primed human peripheral blood monocytes or murine macrophages coincubated with either PBS, Ad‐MSCs (2.5 × 10^5^ cells), or transfected with miR‐466 mimics (10 μM) were collected, respectively, stained with surface markers such as CD14, CD68, CD86, CD206, F4/80, CD11b, and CD16/32. Intracellular markers, such as CD68, CD86, and CD206, were also stained according to the manufacturer's instructions. The detailed information is described in Supporting Information. Human peripheral blood monocytes were characterized by positive staining for CD14. CD206 was one of the markers of the type 2 macrophages, whereas CD86 was used to represent type 1 macrophages derived from human cells. On the other hand, macrophages were characterized by positive staining for CD16/32 and F4/80 with CD206 positive for M2 and CD16/32 positive for M1 in mice derived macrophages. All the samples were obtained by CytExpert and analyzed with flowjo Software.

#### 2.2.3 MicroRNA array and measurement of microRNA expression

Total RNA for exploring microRNA expression was extracted using the miRNeasy Mini Kit (Qiagen, Valencia, CA) according to the manufacturer's instructions. RNA was eluted in RNase‐free water and stored at −80°C. MicroRNA array was performed by Aksomics Inc. (Shanghai, China) using two batches of Ad‐MSC EVs isolated from different dates. The expression of microRNA was conducted on miScript II RT Kit and miScript SYBR Green PCR Kit (Qiagen). The separate well 2^−ΔΔCt^ cycle threshold method was used to determine relative quantitative levels of individual microRNA and these were expressed as the fold difference to the *RUN6*. The microRNA‐specific miScript primer assays were purchased from Qiagen and inventory of involved primers was listed as follows: *RUN6* (Cat#MS00033740), miR‐103‐2‐5p (Cat#MS00043386), miR‐344h‐3p (Cat#MS00042679), miR‐107 (Cat#MS00032235), miR‐144 (Cat#MS00024213), miR‐26a (Cat#MS00032613), miR‐302a (Cat#MS00011690), miR‐26b (Cat#MS00011557), miR‐101a (Cat#MS00011011), and miR‐142‐5p (Cat#MS00006062).

#### 2.2.4 Transfection with microRNA mimics

MicroRNA mimic negative control#1 (NS‐m, Cat#4464058) and miR‐466 mimics (miR‐466‐m, ID: MC20013, Cat#MIMAT0017325) were obtained from Ambion (Thermo Fisher Scientific, http://www.thermofisher.com). Ad‐MSCs or macrophages (PMs and RAW264.7 cells) were transfected with microRNA using Lipofectamine™ RNAiMAX Transfection Reagent (Invitrogen, Thermo Fisher Scientific) following the manufacturer's instructions (Table S1).

#### 2.2.5 Confocal microscopy

Macrophages were exposed to GFP‐PA in a MOI of 100:1 for 1 h, and incubated with primary anti‐alpha Tubulin antibody (ab7291; Abcam, Cambridge, UK; https://www.abcam.com) overnight at 4°C before exposure to the secondary cross‐adsorbed antibody (Goat anti‐Mouse IgG (H+L) (Alexa Fluor 555, Invitrogen, Thermo Fisher Scientific) for 1 h at room temperature. Cells were lastly incubated with DAPI, known as a semipermeant/impermeant nucleic acid stain (Beyotime, Shanghai, China; http://www.beyotime.com) for 20 min to stain the nucleus.

Ad‐MSCs EVs were labeled fluorescently with the cell membrane dye, Dil (Sigma), prior to incubation with RAW264.7. After incubation for 12 h, RAW 264.7 was washed and fixed by 4% paraformaldehyde and stained with DAPI. RAW264.7 cells were transfected with FAM‐labeled miR‐466 mimics using Lipofectamine RNAiMAX Transfection Reagent (Invitrogen, Thermo Fisher Scientific) following the manufacturer's instructions (Table S1) and then were stained with DAPI 24 h later. Images were obtained using the DeltaVision OMX microscope (GE Healthcare Life Sciences, CT).

#### 2.2.6 Phagocytosis of macrophages

RAW264.7 cells (5 × 10^5^ cells/well) were primed with or without Ad‐MSCs (Transwell plates, 2.5 × 10^5^ cells in the upper chamber, 0.4 μm pore size, Costar, Corning; http://www.corning.com/asean/en/products/life-sciences.html) or miR‐466 mimics (10 μM) for 24 hours before exposure to MDR‐PA (MOI 1:1). After 1 h, gentamicin (100 μg/ml) was added for another 1 h to kill bacteria outside of the cells and 1 ml 1% SDS was then added for permeabilization. After 10 min, the supernatant was collected and plated on LB agar plate for CFU counts. GFP‐PA primed RAW264.7 macrophage cells coincubated with either PBS, Ad‐MSCs (2.5 × 10^5^ cells) or miR‐466 mimics (10 μM) were collected, respectively, washed with cold DPBS once, and subjected to BD Accuri C6 Flow Cytometer according to the manufacturer's instructions.

#### 2.2.7 Luciferase reporter assay

To identify the molecular target of miR‐466, we searched for predicted miR‐466 targets using bioinformatics tools, focusing our attention on the regulators of inflammation. In agreement with this, the public database of the microRNA.org (miRanda) lists Toll/interleukin‐1 receptor domain‐containing adaptor protein (TIRAP) as one of the potential targets of miR‐466. For luciferase reporter assays, *TIRAP* 3′UTR normal and mutant constructs (50 ng/well) were cotransfected with miR‐466 mimics (50 nM) or nontarget control (50 nM) in 293T cells using Lipofectamine 3000 (Invitrogen, Thermo Fisher Scientific). After 48 h of transfection, cells were added to luciferase substrate and luciferase activity was measured using the Luciferase illuminometer.

#### 2.2.8 Murine model of pneumonia induced by intratracheal inoculation with multidrug‐resistant PA

Mice were inoculated intratracheally (IT) with 10^6^ CFU of MDR‐PA strain and were then instilled IT with 40 μl of either PBS, Ad‐MSCs (8 × 10^5^), Ad‐MSC EVs, miR‐466‐m‐MSC EVs, or L‐929 EVs as a negative control 4 h later (described in Supporting Information). In another separate experiment, mice were injected with antagomiR‐466 (RiboBio, Guangzhou, China) via tail veins at a dose of 10 mg/kg of body weight in 0.2 mL saline 24 h before inoculated IT with PA. A scramble antagomiR (anta‐Ctl) was used as a control. Then the mice received the same treatment and the samples as describe below were collected.

Twenty‐four hours following the injury, bronchoalveolar lavage fluid (BALF), blood samples, or lungs were collected. Total cell count and differential were obtained using Sysmex pocH‐100i (Sysmex Shanghai Ltd., Shanghai, China) according to the manufacturer's protocols. Macrophage inflammatory protein (MIP)‐2, which is known as a mouse neutrophil chemokine, and tumor necrosis factor‐α (TNF‐α) were measured by ELISA (R&D Systems, Minneapolis, MN) in the BALF supernatants. BALF protein concentration was also measured using BCA Protein Assay Kit (Beyotime http://www.beyotime.com/). Quantitative bacterial analysis and bacterial strain identification were performed on BALF and lung homogenate using techniques detailed in the online data supplement. Following sacrifice, lungs were excised and prepared for histologic examination as detailed in the Supporting Information. An independent investigator evaluated whether lung parenchyma was infected or not. We defined pneumonia as the presence of polymorphonuclear neutrophils within septa and alveolar spaces.^28^ The severity of lung injury was determined according to the scoring system of the American Thoracic Society.^29^

#### 2.2.9 Analysis

Results are expressed as mean ± standard error of mean (SEM) if the data were normally distributed and median (interquartile range [IQR]) if not. Comparisons between two groups were analyzed using unpaired *t*‐test or Mann–Whitney test. Comparisons between more than two groups were analyzed using an analysis of variance (ANOVA) or Kruskal–Wallis test using the Bonferroni's correction for multiple‐comparison testing. The log‐rank test was used for comparing survival rate at 96 h. A *p* value < .05 was considered statistically significant. All statistical analysis was performed using GraphPad Prism software (La Jolla, CA). *N* in the figure legends refers to the number of samples or mice and is not the number of replicate experiments of the same sample or mouse.

## 3 RESULTS

### 3.1 Expression of miR‐466 in Ad‐MSC EVs

**FIGURE 1:**
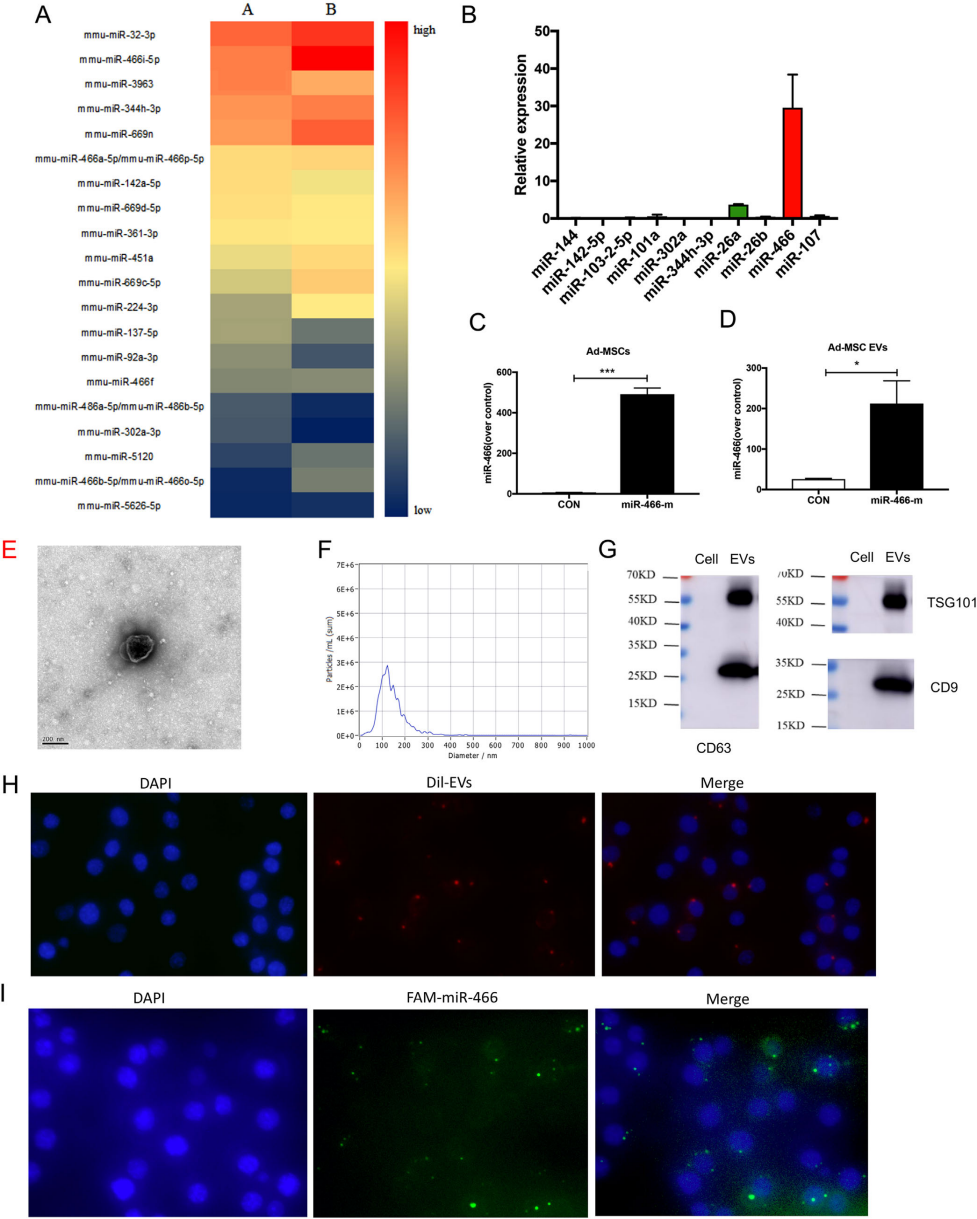
(A) The heat‐map presents the first 20 most expressed microRNAs in Ad‐MSC EVs. (B) qRT‐PCR was used to validate the result from microRNA array. (C) Twenty‐four hours following the transfection with miR‐466 mimics, the expression of miR‐466 was significantly higher in Ad‐MSCs than those transfected with NS‐m (491.4 ± 17.5 vs. 4.2 ± 2.1, *p* < .001, *N* = 4). (D) The expression of miR‐466 in EVs derived from miR‐466 mimics transfected Ad‐MSCs was significantly higher than those derived from Ad‐MSCs transfected with NS‐m (25.9 ± 1.2 vs. 212.3 ± 32.4, *p *< .05, *N* = 4). (E) Representative electron microscopic photographs of Ad‐MSC EVs, scale bar = 200 nm. (F) Concentration and size distribution of Ad‐MSC EVs were determined by NTA. (G) Representative western blots showing the expression of exosome markers, including CD63, CD9, and Tsg101. (H) Ad‐MSC EVs were labeled with Dil (red) and then incubated with RAW 264.7 cells. DAPI (blue) was used to stain the nucleus. (I) RAW 264.7 cells were transfected with FAM‐labeled miR‐466 mimics (green) for 24 h. DAPI (blue) was used to stain the nucleus. MiR, microRNA; Ad‐MSC, mouse adipose‐derived mesenchymal stromal cells; Ad‐MSC EV, mouse adipose‐derived mesenchymal stromal cells derived extracellular vesicles; CON, control

For the sake of the optimal microRNAs in Ad‐MSC EVs, the selection of 20 of the highest expressed microRNAs among more than 3000 has been based on the heat map of two batches of EVs isolated from different dates (Figure 1A and Table S4). We subsequently determined the expression of the 10 most intensely expressed microRNAs using quantitative real time PCR (qRT‐PCR) (Figure 1B) to validate the results from the microRNA array. Both microRNA array and qRT‐PCR indicated that miR‐466 was highly expressed in Ad‐MSC EVs. When Ad‐MSCs transfected with miR‐466 mimics, the expression of miR‐466 was 400‐fold higher in Ad‐MSCs (Figure 1C) and 80‐fold higher in Ad‐MSC EVs (Figure 1D).

### 3.2 Characterization of EVs derived from Ad‐MSCs

The characterization of EVs derived from Ad‐MSCs was also measured. As shown in Figure 1E, the morphologies of EVs were observed by transmission electron microscopy examination. The results of NTA showed that the size distribution of EVs was 50–400 nm in diameter and the concentration of EVs in our experiments equals 5.5 × 10^7^ particles/ml (Figure 1F). The EVs were enriched in the specific markers of exosome, including Tsg101, CD63, and CD9 (Figure 1G), suggesting that the majority of EVs were likely exosomes derived from Ad‐MSCs.

To see the endocytosis of macrophages to Ad‐MSC EVs, we used Dil, a fluorescent dye, to label the Ad‐MSC EVs. As shown in Figure 1H, the red fluorescence clearly presented within RAW 264.7 following incubation with Dil‐labeled EVs for 12 h. FAM was used to label miR‐466 mimics. Similarly presented in Figure 1I, the green fluorescence clearly presented within RAW 264.7 following transfected with FAM labeled miR‐466 mimics for 24 h.

### 3.3 Effect of human MSCs in promoting human macrophage polarization toward type 2 phenotype

**FIGURE 2:**
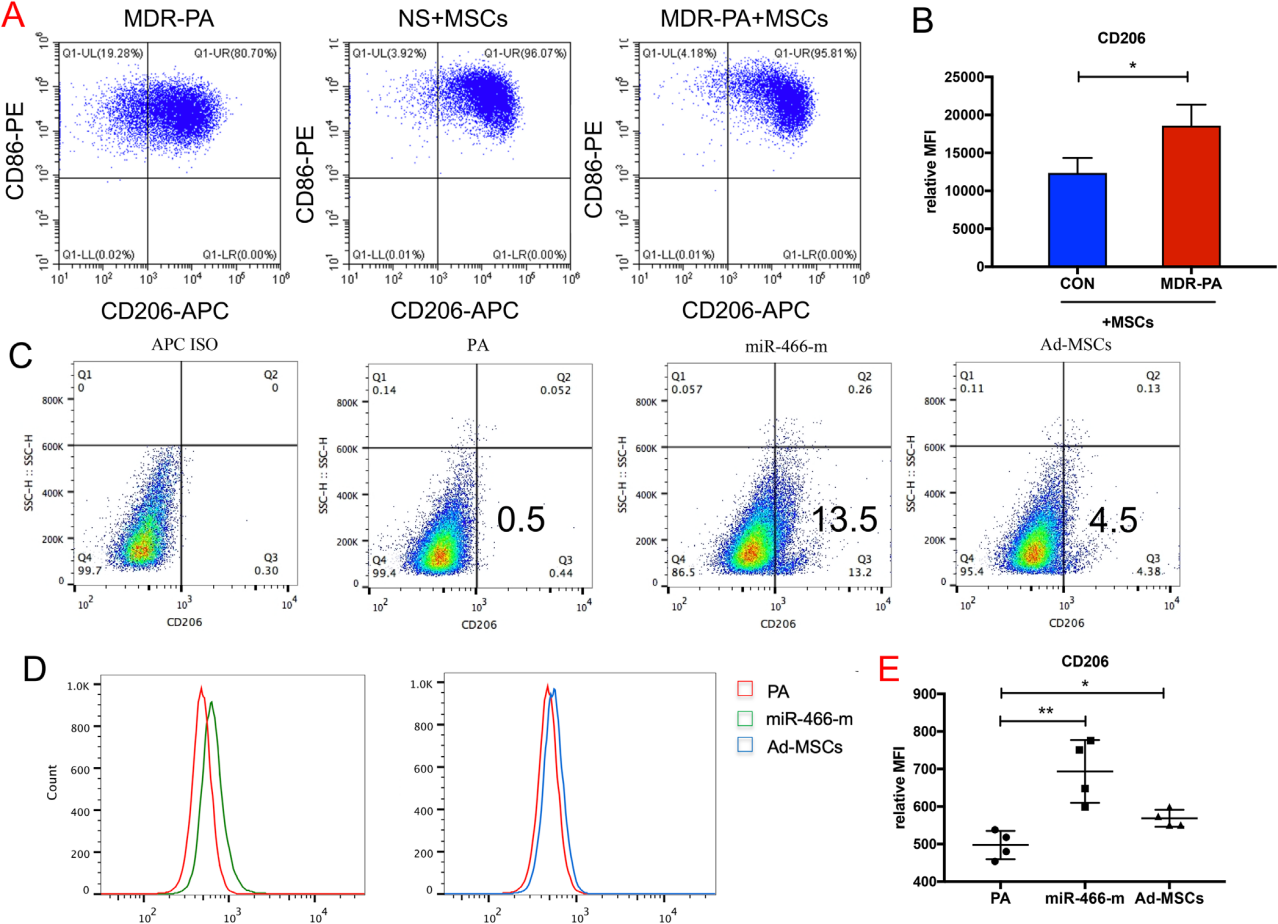
When human peripheral blood monocytes were cocultured with h‐MSCs (A and B). The MFI was significantly increased in human peripheral blood monocytes (*p < .05, *p *= .034, 12 359 ± 1147 for healthy people vs. 18 586 ± 1601 for MDR‐PA patients, *N* = 4). When macrophages were cocultured with Ad‐MSCs or transfected with the miR‐466‐m prior to the presence of MDR‐PA, (C and D) statistically significant higher expressions in the proportion of CD206 positive cell population (13.7 ± 0.7% for miR‐466‐m, 4.9 ± 0.3% for Ad‐MSCs, 0.5 ± 0.04% for PA, *p *< .001 for PA vs. Ad‐MSCs, *p *< .001 for PA vs. miR‐466‐m, *N* = 9). (E) The MFI was significantly increased in RAW264.7 (**p < .01, *p *= .005, 497.5 ± 18.8 for PA vs. 693.5 ± 41.9 for miR‐466‐m, *p* = .02, 497.5 ± 18.8 for PA vs. 568.8 ± 11.45 for Ad‐MSCs, *N* = 9). PA, *Pseudomonas aeruginosa*; Ad‐MSC, mouse adipose‐derived mesenchymal stromal cells; MiR‐466‐m, Ad‐MSCs transfected with miR‐466‐mimics; APC ISO, allophycocyanin isotype; CD206, cluster of differentiation 206

We evaluated the role played by MSCs in macrophage polarization by flow cytometry analyses. After 2 days of coculture with h‐MSCs, CD14‐positive human monocytes derived from peripheral blood of patients with MDR‐PA‐induced pneumonia expressed high levels of CD206 significantly, compared to those monocytes derived from healthy people, as determined by MFI (*p *= .034, Figure 2A and B).

### 3.4 Effect of Ad‐MSCs and miR‐466 on macrophages

We further evaluated the role played by miR‐466 in macrophage polarization by flow cytometry analyses. When macrophages were co‐cultured with Ad‐MSCs or transfected with the miR‐466 mimics prior to exposure to MDR‐PA, we observed a significant increase in the proportion of CD206 positive cell population (*p *< .001) as well as in the mean fluorescence intensity (MFI) of expression of this specific marker of the macrophage type 2 phenotype (Figure 2C‐E). Macrophages transfected with the miR‐466‐m showed remarkable increases in expression of mRNA coding for M2‐related *Arg‐1* (by 78%) and *IL‐10* (by 67%), but a significant decrease in those coding for M1‐related *iNOS* (by 55%) and *IL‐12* (by 58%) (Figure S2). In separate experiments, similar findings were observed in primary peritoneal macrophages (Figure S2).

**FIGURE 3:**
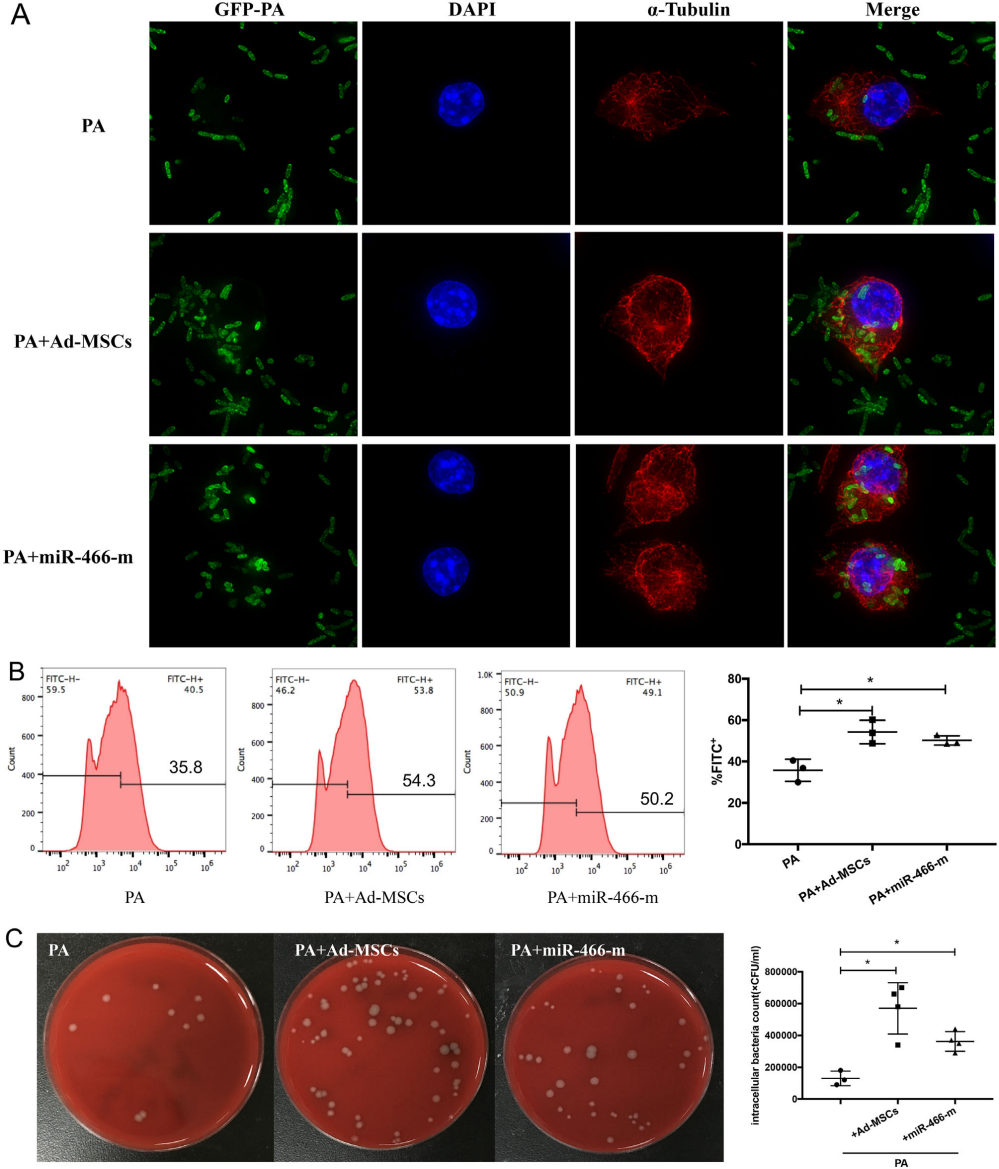
(A) When cocultured with Ad‐MSCs, fluorescent confocal imaging showed significantly more intracellular bacteria within macrophages. Similar findings were observed when macrophages were transfected with miR‐466‐m. DAPI (blue) was used to stain the nucleus. Anti‐alpha Tubulin antibody (red) was used to stain the cytoskeleton. The green represented GFP‐PA. (B) Flow cytometry analyses suggested an increased phagocytosis when macrophages were cocultured with Ad‐MSCs or transfected with miR‐466‐m (*p* = .01, *p < .05, 54.3% ± 3.3 for Ad‐MSCs vs. 35.8% ± 3.1 for PA control; *p* = .01, *p < .05, 50.2% ± 1.3 for miR‐466‐m vs. 35.8% ± 3.1 for PA control, *N* = 3 for PA control, *N* = 3 for Ad‐MSCs, *N* = 3 for miR‐466‐m). (C) The CFU counting of intracellular bacteria were significantly increased in either cocultured group or miR‐466‐m transfected group (*p* = .006, **p < .01, 570 000 ± 80 623 for Ad‐MSCs vs. 130 000 ± 26 458 for PA, *p* = .003, **p < .01, 362 500 ± 30 923 for miR‐466‐m vs. 130 000 ± 26 458 for PA, *N* = 3 for PA control, *N* = 4 for Ad‐MSCs, *N* = 4 for miR‐466‐m). PA, *Pseudomonas aeruginosa*; Ad‐MSC, mouse adipose‐derived mesenchymal stromal cells; MiR‐466‐m, Ad‐MSCs transfected with miR‐466‐mimics; GFP, green fluorescent protein; DAPI, 4′,6‐diamidino‐2‐phenylindole

We further explored the effect of miR‐466 on macrophages phagocytosis against MDR‐PA. When cocultured with Ad‐MSCs or transfected with miR‐466‐m, macrophages showed significantly more intracellular bacteria in fluorescent confocal images (Figure 3A and Supporting Information Video) and increased phagocytosis in flow cytometry analyses (Figure 3B). After eliminating extracellular bacteria, the CFU counting of intracellular bacteria was significantly increased in either cocultured group or miR‐466‐m transfected group (Figure 3C).

### 3.5 Transfer of miR‐466‐containing Ad‐MSC EVs from Ad‐MSCs to macrophages impacts the cytokine secretion profile of macrophages

**FIGURE 4:**
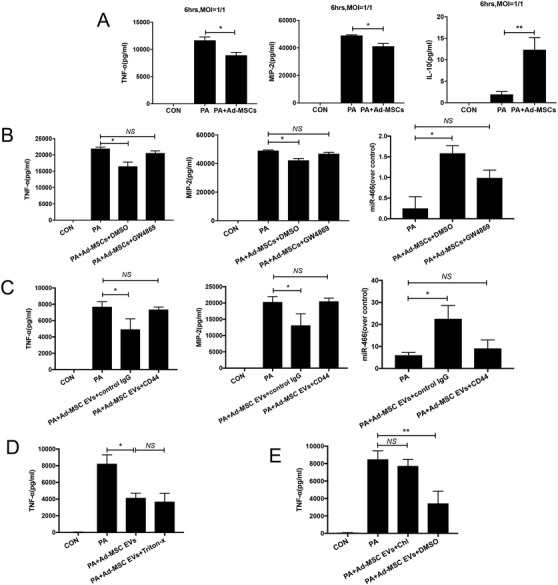
(A) After 6 h of MDR‐PA exposure, TNF‐α (*p* = .03, *p < .05, 8929 ± 532.9 for Ad‐MSCs cocultured group vs. 11 669 ± 620.6 for PA, *N* = 3 for control, PA, and Ad‐MSCs) and MIP‐2 (*p* = .03, *p < .05, 41 207 ± 1999 for Ad‐MSCs cocultured group vs. 49 009 ± 491.7 for PA, *p* = .019, *N* = 3 for control, PA, and Ad‐MSCs) were significantly decreased in RAW264.7 cells cocultured with Ad‐MSCs, while the level of IL‐10 was significantly augmented in the same group (*p* = .005, **indicates *p* < .01, 12.4 ± 2.8 for Ad‐MSCs cocultured group vs. 1.9 ± 0.7 for PA, *N* = 6 for control, PA, and Ad‐MSCs). (B) Incubation with GW4869 prior to MDR‐PA exposure mostly abrogated the reduction of TNF‐α (21 950 ± 434.6 for PA, 16 532 ± 1264 for DMSO control, 20,569 ± 693.7 for GW4869, *p* = .2 for PA vs. GW4869, *p < .05, *p* = .02 for PA vs. DMSO control, *p* = .2 for PA vs. GW4869, *N* = 4 for PA, DMSO control, and GW4869) and MIP‐2 levels of secretion (49 009 ± 491.7 for PA, 42 207 ± 1307 for DMSO control, 46 869 ± 1018 for GW4869, **p < .01, *p* = .008 for PA vs. DMSO control, *p* = .131 for PA vs. GW4869, *N* = 4 for PA, DMSO control, and GW4869). The increased expression of miR‐466 in macrophages was significantly abrogated when either Ad‐MSC EVs secretion was blocked by GW4869 (0.3 ± 0.2 for PA, 1.6 ± 0.1 for DMSO control, 1.0 ± 0.1 for GW4869, *p < .05, *p *= .03 for PA vs. DMSO control, *p *= .09 for PA vs. GW4869, *N* = 4 for PA, DMSO control, and GW4869). (C) RAW 264.7 cells exposure with pretreated Ad‐MSC EVs with blocking anti‐CD44 showed significant reduction in TNF‐α (7691 ± 364.4 for PA, 4928 ± 752.7 for IgG control, 7363 ± 172.6 for CD44, *p < .05, *p* = .03 for PA vs. IgG control, *p* = 0.4606 for PA vs. CD44, *N* = 4 for PA, IgG control, and CD44) and MIP‐2 levels of secretion (20 302 ± 962.9 for PA, 13 144 ± 2036 for IgG control, 2503 ± 572.7 for CD44, *p < .05, *p* = .03 for PA vs. IgG control, *p* = .9 for PA vs. CD44, *N* = 4 for PA, IgG control, and CD44). The increased expression of miR‐466 in macrophages was significantly abrogated when Ad‐MSC EVs uptake was blocked by an anti‐CD44 (6.0 ± 0.9 for PA, 22.6 ± 3.5 for IgG control, 9.1 ± 2.2 for CD44, *p < .05, *p *= .04 for PA vs. IgG control, *p* = .4 for PA vs. CD44, N = 4 for PA, IgG control, and CD44). (D) RAW 264.7 cells exposure with pretreated Ad‐MSC EVs with Triton‐X100 showed no significant difference in TNF‐α (8241 ± 615.0 for PA, 4928 ± 752.7 for PA+Ad‐MSC EVs, 3692 ± 584.2 for PA+Ad‐MSC EVs+Triton‐X100, **p < .01, *p* = .0042 for PA vs. PA+Ad‐MSC EVs, *p* = .5340 for PA+Ad‐MSC EVs vs. PA+Ad‐MSC EVs+Triton‐X100, *N* = 4 for PA, PA+Ad‐MSC EVs and PA+Ad‐MSC EVs+Triton‐X100). (E) Pretreating RAW264.7 cells with chlorpromazine hydrochloride caused that the decrease of TNF‐α levels was abrogated (8495 ± 564.4 for PA, 7730 ± 436.9 for PA+Ad‐MSC EVs+Chl, 7363 ± 172.6 for PA+Ad‐MSC EVs+DMSO, *p* = .334 for PA vs. PA+Ad‐MSC EVs+Chl, ** indicates *p* < .01, *p* = .0068 for PA+Ad‐MSC EVs+Chl vs. PA+Ad‐MSC EVs+DMSO, *N* = 4 for PA, PA+Ad‐MSC EVs+Chl and PA+Ad‐MSC EVs+DMSO), confirming that functional Ad‐MSC EVs were more likely taken up by endocytosis. TNF‐α, tumor necrosis factor‐α; MIP‐2, macrophage inflammatory protein 2; IL‐10, interleukin 10; PA, *Pseudomonas aeruginosa*; Ad‐MSC, mouse adipose‐derived mesenchymal stromal cells; Ad‐MSC EV, mouse adipose‐derived mesenchymal stromal cells derived extracellular vesicles; CON, control; MOI, multiplicity of infection; DMSO, dimethyl sulfoxide; IgG, immunoglobulin G; CD44, cluster of differentiation 44

When MDR‐PA‐exposed macrophages were cocultured with Ad‐MSCs, we observed a significant decrease in TNF‐α and MIP‐2 levels while the level of IL‐10 was significantly augmented (Figure 4A). We subsequently found that incubation with GW4869 (10 μM) prior to MDR‐PA exposure mostly abrogated the reduction of TNF‐α (by 20%) and MIP‐2 levels of secretion, as well as the expression of miR‐466 in macrophages (Figure 4B). Similarly, pretreating Ad‐MSC EVs with blocking anti‐CD44 (to block cells uptake of Ad‐MSc EVs) showed similar suppression of the reduction in TNF‐α and MIP‐2 levels of secretion and expression of miR‐466 (Figure 4C).

We then pretreated Ad‐MSC EVs with Triton‐X100 before incubation with MDR‐PA‐exposed macrophages, and TNF‐α levels showed no significant increase in the pretreating group (Figure 4D), demonstrating that these regulatory functions were EV specific and not due to any coisolated proteins.

We have duplicated the experiments using Chlorpromazine hydrochloride, which is well known as blocking endocytosis, the decrease of TNF‐α levels were abrogated (Figure 4E), confirming that functional Ad‐MSC EVs were more likely taken up by endocytosis.

### 3.6 Role of miR‐466 on TIRAP‐MyD88‐NFkB axis

**FIGURE 5:**
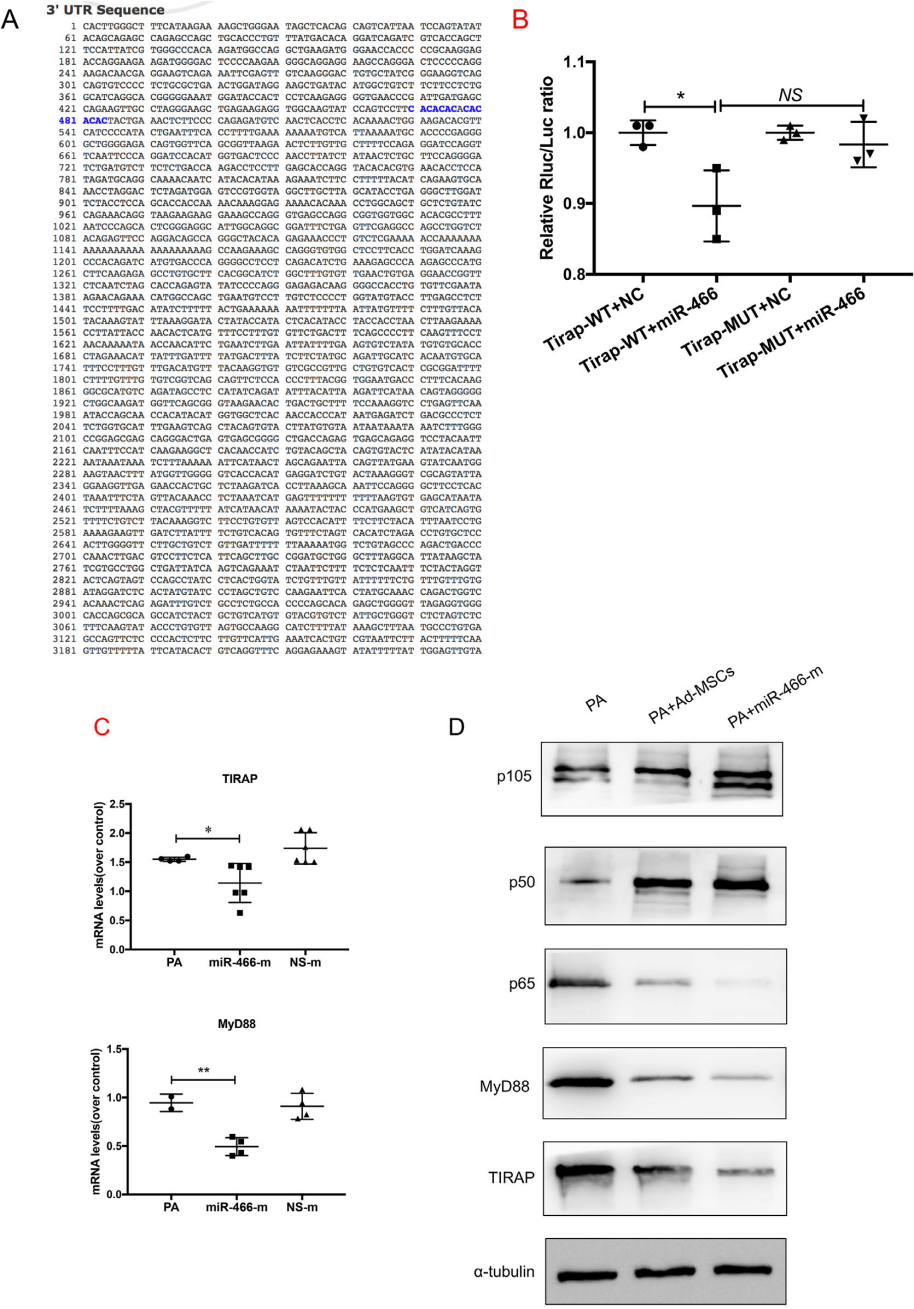
(A) TIRAP mRNA has a conservative miR‐466 sequence in its 3′UTR 470–484 position (highlighted in blue). (B) Luciferase reporter assay showed that the relative Rluc/Luc ratio was significantly decreased when TIRAP‐WT vector was bound by miR‐466 (*p* = .03, *p < .05, 0.8 ± 0.05 vs. 1.0 ± 0.01). (C) qRT‐PCR showed that TIRAP (*p* = .04, *p < .05, 1.6 ± 0.02 for PA vs. 1.1 ± 0.1 for miR‐466‐m, *N* = 4) and MyD88 (*p* = .005, **p < .01, 0.9 ± 0.06 for PA vs. 0.5 ± 0.05 for miR‐466‐m, *N* = 4) were significantly reduced in cells transfected with the miR‐466‐m. (D) Western blot showed a decreased in protein expression of TIRAP, MyD88, and p65, while the expression of p105 and p50, key proteins involved in NF‐κB pathway, was augmented. PA, *Pseudomonas aeruginosa*; Ad‐MSC, mouse adipose‐derived mesenchymal stromal cells; MiR‐466‐m, Ad‐MSCs transfected with miR‐466‐mimics; NS‐m, Ad‐MSCs transfected with negative mimics; EV, extracellular vesicles; TIRAP, Toll/interleukin‐1 receptor domain‐containing adaptor protein; MyD88, myeloid differentiation primary response 88; WT, wild type; NC, negative control; MUT, mutation; Rluc, renilla luciferase; Luc, luciferase; NF‐κB, nuclear factor κB

To identify the molecular target of miR‐466, we sought out for predicted miR‐466 targets using bioinformatics tools (miRanda, TargetScan, PicTar, RNA22, and miRDB). Taking into account as a priority the targets including inflammation regulators, *TIRAP* was supposed to be one of the potential targets of miR‐466. *TIRAP* mRNA has a conservative miR‐466 seed sequence in its 3′UTR 470–484 region (Figure 5A). Luciferase reporter assay showed that the relative Rluc/Luc ratio was significantly decreased when TIRAP‐WT vector was bound by miR‐466 (0.8 ± 0.05 vs. 1.0 ± 0.01, *p *= .03) (Figure 5B), corroborating that miR‐466 specifically targeted a seed region of mRNA coding for TIRAP, leading to decreased TIRAP gene expression. Expression of both *TIRAP* and *MyD88* mRNA was significantly reduced in RAW264.7 cells transfected with the miR‐466 mimics (Figure 5C) and accompanied with a decreased protein expression of TIRAP and MyD88. Moreover, miR‐466 significantly inhibited the expression of p65, while the expression of p105 and p50, key proteins involved in NF‐κB pathway, was augmented (Figure 5D).

### 3.7 Mortality of the experimental model and dose–response effect of intratracheal Ad‐MSCs and Ad‐MSC EVs

The optimal therapeutic effect was obtained with the IT administration of 8 × 10^5^ Ad‐MSCs and Ad‐MSC EVs (Figures S3 and S4). Ad‐MSCs and Ad‐MSC EVs had similar therapeutic effects as h‐MSCs and h‐MSC EVs (Figure S5).

**FIGURE 6:**
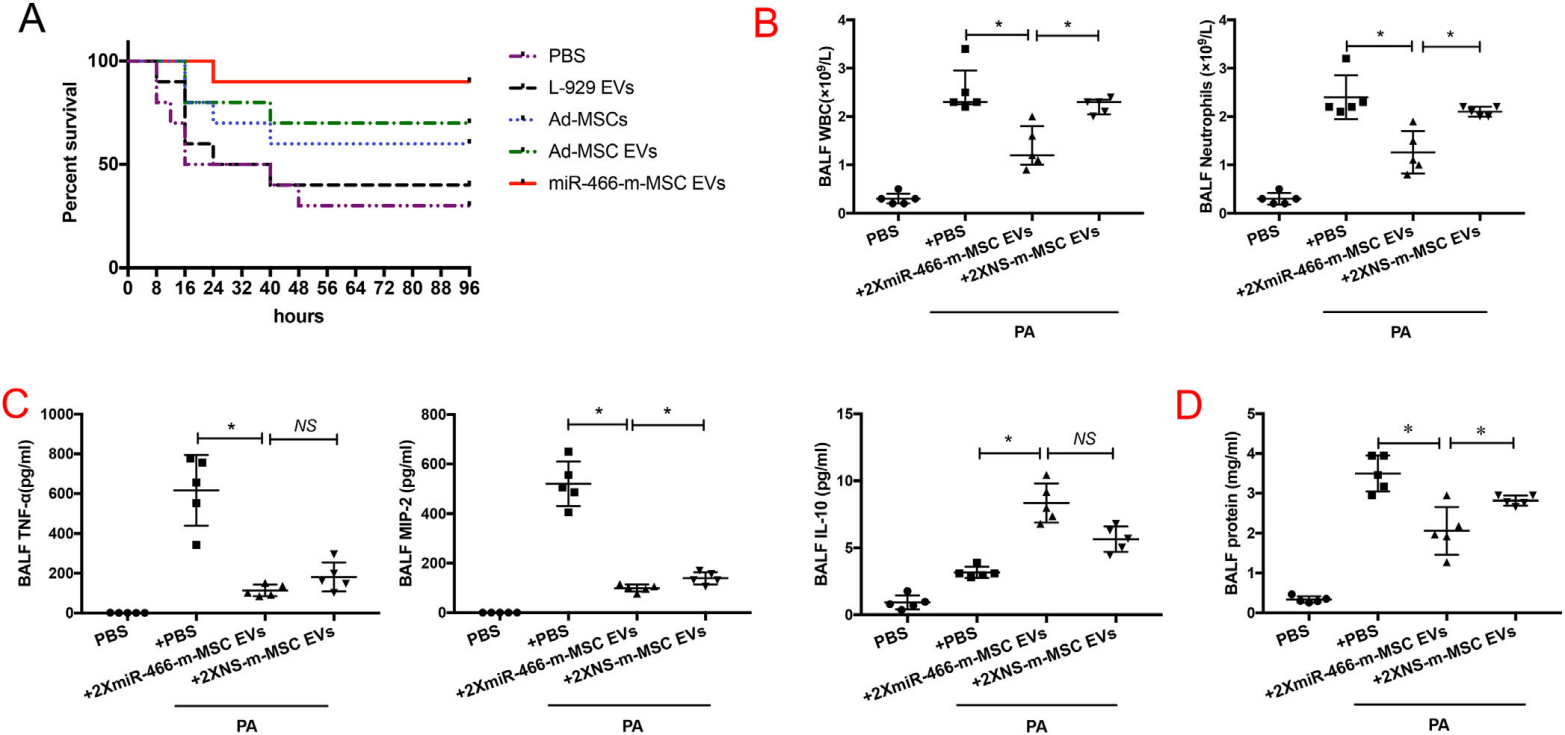
(A) Kaplan–Meier survival curves of MDR‐PA infected C57BL/6 mice treated by different ways. IT instillation of Ad‐MSC EVs or miR‐466‐m‐MSC EVs significantly increased survival over 96 h. (*p *< .05 for Ad‐MSC EVs vs. PBS, *p *< .001 for miR‐466‐m‐MSC EVs vs. PBS, *N *= 10 for each group). (B) IT administration of miR‐466‐m‐MSC EVs postinfection decreased the influx of white cells (*p* = .04, *p < .05, 1.4 ± 0.2 for miR‐466‐m‐MSC EVs, 2.2 ± 0.1 for NS‐m‐MSC EVs, *N* = 5 for PA, *N* = 5 for miR‐466‐m‐MSC EVs, *N* = 5 for NS‐m‐MSC EVs) and neutrophils (*p* = .04, *p < .05, 1.3 ± 0.2 for miR‐466‐m‐MSC EVs, 2.1 ± 0.1 for NS‐m‐MSC EVs, *N* = 5 for PA, *N* = 5 for miR‐466‐m‐MSC EVs, *N* = 5 for NS‐m‐MSC EVs) in BALF at 24 h as compared with either NS‐m‐MSC EVs groups. (C) The BALF levels of TNF‐α (*p* = .02, ** indicates *p* < .05, 124.8 ± 20.4 for miR‐466‐m‐MSC EVs vs. 198.7 ± 55.8 for NS‐m‐MSC EVs, *N* = 5 for PA, *N* = 5 for miR‐466‐m‐MSC EVs, *N* = 5 for NS‐m‐MSC EVs), MIP‐2 (*p* = .04, **p < .05, 95.2 ± 9.0, *p* = .04 for miR‐466‐m‐MSC EVs vs. 144.5± 12.9 for NS‐m‐MSC EVs, *N* = 5 for PA, *N* = 5 for miR‐466‐m‐MSC EVs, *N *= 5 for NS‐m‐MSC EVs) were decreased in the miR‐466‐m‐MSC EVs group. The level of IL‐10 expression was increased (*p* = .004, **p < .01, 8.2 ± 1.0 for miR‐466‐m‐MSC EVs vs. 3.3 ± 0.2 for PA, *N* = 5 for PA, *N* = 5 for miR‐466‐m‐MSC EVs, *N* = 5 for NS‐m‐MSC EVs) when compared with other groups. (D) Total protein concentration was decreased in miR‐466‐m‐MSC EVs group (*p* = .03, *p < .05, 1.8 ± 0.3 for miR‐466‐m‐MSC EVs vs. 2.7 ± 0.04 for NS‐m‐MSC EVs, *N* = 5 for PA, *N* = 5 for miR‐466‐m‐MSC EVs, *N* = 5 for NS‐m‐MSC EVs). BALF, bronchoalveolar lavage fluid; WBC, white blood cell; TNF‐α, tumor necrosis factor‐α; MIP‐2, macrophage inflammatory protein 2; IL‐10, interleukin 10; PA, *Pseudomonas aeruginosa*; MiR‐466‐m‐MSC EVs, extracellular vesicles derived from Ad‐MSCs transfected with miR‐466 mimics; NS‐m‐MSC EVs, extracellular vesicles derived from Ad‐MSCs transfected with miR‐466 mimics negative control

Increasing MDR‐PA inoculum increased 4‐day mortality. IT 5 × 10^6^ CFU of MDR‐PA resulted in significant inoculation lethal pneumonia, leading to 30% survival at 96 h (Figure S7A). IT Ad‐MSC EVs increased survival to 70% at 96 h compared with mice treated with PBS or L‐929 EVs as negative controls. Instillation of miR‐466‐m‐MSC EVs further increased survival to 90% at 96 h. Significant difference was found between the survival of miR‐466‐m‐MSC EVs group and PBS group (*p *< .01), and between the survival of miR‐466‐m‐MSC EVs group and L‐929 EVs group (*p* = .02) (Figure 6A).

### 3.8 Therapeutic effects of Ad‐MSC EVs derived from Ad‐MSCs transfected with miR‐466 mimics (miR‐466‐m‐MSC EVs) on lung inflammation, protein permeability, and histological severity in MDR‐PA inoculation pneumonia

IT administration of 10^6^ CFU MDR‐PA induced nonlethal but a robust pneumonia‐induced acute lung injury over 24 h, characterized by an influx of white blood cells, neutrophils, high levels of TNF‐α and MIP‐2, increased lung protein permeability, and development of bacterial load in BALF (Figures S6 and S7).

**FIGURE 7:**
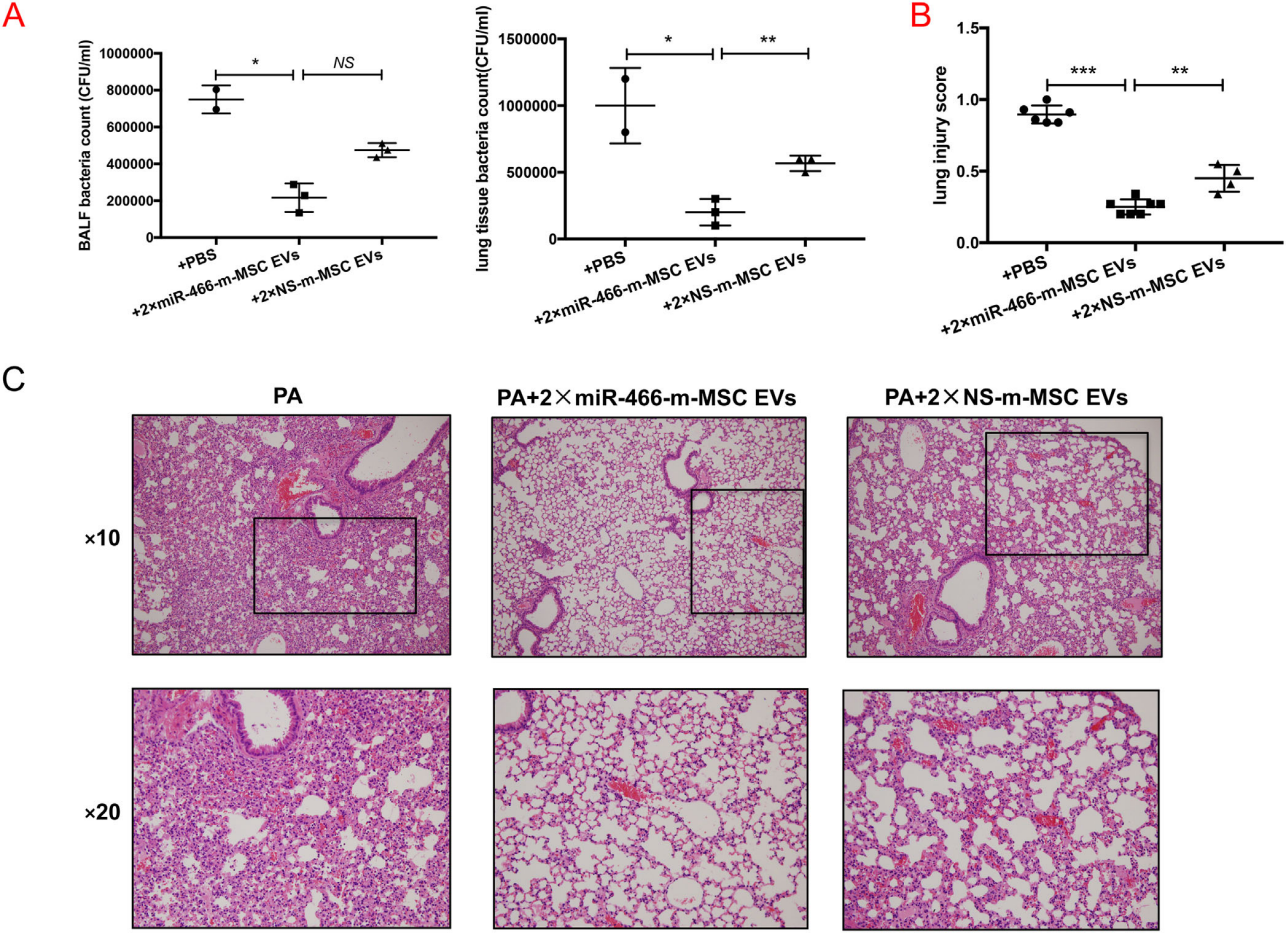
(A) IT instillation of miR‐466‐m‐MSC EVs significantly decreased the bacterial load in both BALF (*p* = .03, *p < .05, 211 500 ± 76 500 for IT instillation of miR‐466‐m‐MSC EVs vs. 750 000 ± 54 000 for PA; *p* = .07, 211 500 ± 76 500 for IT instillation of miR‐466‐m‐MSC EVs vs. 494 000 ± 18 000 for NS‐m‐MSC EVs, *N* = 3 for PA, *N* = 4 for miR‐466‐m‐MSC EVs, *N* = 4 for NS‐m‐MSC EVs) and lung homogenate (*p* = .02, *p < .05, 200,000 ± 57,735 for IT instillation of miR‐466‐m‐MSC EVs vs. 1 000 000 ± 200 000 for PA; *p* = .005, **p < .01, 200 000 ± 57 735 for IT instillation of miR‐466‐m‐MSC EVs vs. 566 667 ± 33 333 for NS‐m‐MSC EVs, *N* = 3 for PA, *N* = 4 for miR‐466‐m‐MSC EVs, *N* = 4 for NS‐m‐MSC EVs) when compared with either PBS or 2 × NS‐m‐MSC EVs. (B) Treatment with miR‐466‐m‐MSC EVs also reduced histological severity of lung injury better than other groups (*p* < .001, ***p < .001, 0.3 ± 0.02 for miR‐466‐m‐MSC EVs vs. 0.9 ± 0.03 for PA, *p* = .001, **p < .01, 0.5 ± 0.05 for NS‐m‐MSC EVs, *N* = 6 for PA, *N* = 7 for miR‐466‐m‐MSC EVs, *N* = 4 for 90iNS‐m‐MSC EVs). (C) The histology showed less inflammatory cells infiltrating interalveolar septa and respecting alveolar space and lung architecture. BALF, bronchoalveolar lavage fluid; PA, *Pseudomonas aeruginosa*; miR‐466‐m‐MSC EVs, extracellular vesicles derived from Ad‐MSCs transfected with miR‐466 mimics; NS‐m‐MSC EVs, extracellular vesicles derived from Ad‐MSCs transfected with miR‐466 mimics negative control

IT administration of miR‐466‐m‐MSC EVs 4 h postinfection decreased the influx of white cells (by 46%) and neutrophils (by 46%) in BALF at 24 h as compared with either PBS or NS‐m‐MSC EVs groups (Figure 6B). The BALF levels of TNF‐α (124.8 ± 20.4), MIP‐2 (95.2 ± 9.0), and total protein concentration (1.8 ± 0.3) were decreased in the miR‐466‐m‐MSC EVs group, while the level of IL‐10 expression was increased (8.2 ± 1) when compared with other groups (Figure 6C and D). Although miR‐466‐m‐MSC EVs treatment reduced blood white blood cells by 36% and blood circulating neutrophils by 40%, the difference was not statistically significant (Figure S8). IT instillation of miR‐466‐m‐MSC EVs significantly decreased the bacterial load in BALF as well as lung homogenate when compared with either PBS or 2 × NS‐m‐MSC EVs (*p* = .03, *p* = .005, Figure 7A). Treatment with miR‐466‐m‐MSC EVs also reduced histological severity of lung injury better than other groups (*p *= .001) (Figure 7B and C).

**FIGURE 8:**
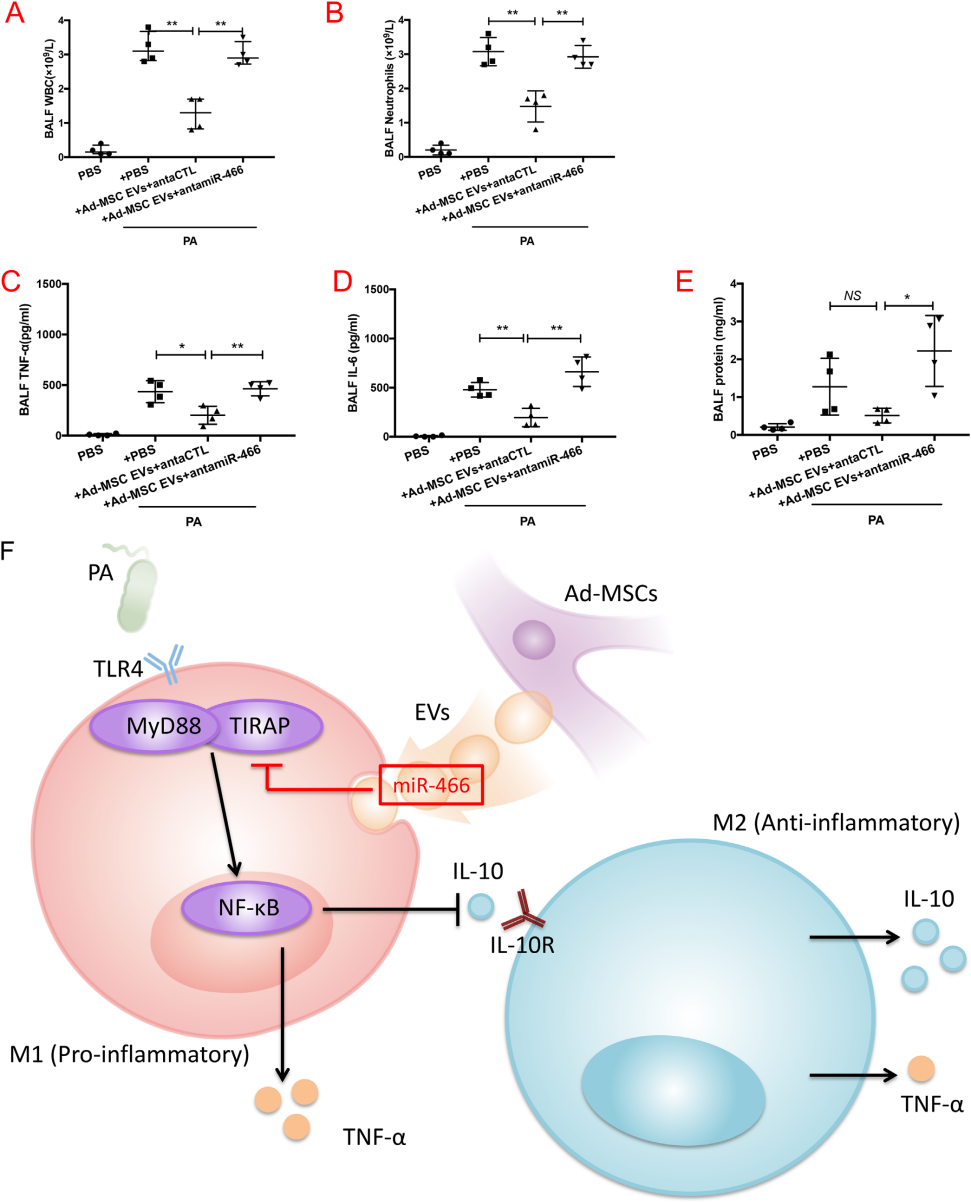
Mice were pretreated with antagomiR‐466 intravenously 24 h before exposed to MDR‐PA. (A and B) pretreated with antagomiR‐466 increased the influx of white cells (*p* = .0013, **p < .01, 3.0 ± 0.2 for antagomiR‐466, 1.5 ± 0.2 for anta‐Ctl, *N* = 5) and neutrophils (*p* = .0021, **p < .01, 2.9 ± 0.2 for antagomiR‐466, 1.3 ± 0.2 for anta‐Ctl, *N* = 5) in BALF. (C and D) The BALF levels of TNF‐α (*p* = .0037, **p < .01, 463.3 ± 35.2 for antagomiR‐466 vs. 202.1 ± 44.4 for anta‐Ctl, *N* = 5) and IL‐6 (*p* = .0019, **p < .01, 662.1 ± 74.9 for antagomiR‐466 vs. 195.7 ± 46.7 for anta‐Ctl, *N* = 5) were increased in antagomiR‐466 group. (E) Total protein concentration was increased in antagomiR‐466 group (*p* = .0118, *p < .05, 2.2 ± 0.5 for antagomiR‐466 vs. 1.7 ± 0.5 for anta‐Ctl, *N* = 5). (F) The diagram shows our main idea that the transfer of miR‐466 from Ad‐MSC EVs to macrophages inhibited TIRAP expression leading to downregulate MyD88‐NF‐κB axis, and therefore, tilting the balance toward M2 polarization. BALF, bronchoalveolar lavage fluid; WBC, white blood cell; TNF‐α, tumor necrosis factor‐α; MIP‐2, macrophage inflammatory protein 2; IL‐10, interleukin 10; PA, *Pseudomonas aeruginosa*; MiR‐466‐m‐MSC EVs, extracellular vesicles derived from Ad‐MSCs transfected with miR‐466 mimics; NS‐m‐MSC EVs, extracellular vesicles derived from Ad‐MSCs transfected with miR‐466 mimics negative control. TIRAP, Toll/interleukin‐1 receptor domain‐containing adaptor protein; MyD88, myeloid differentiation primary response 88; TLR4, toll‐like receptor 4; NF‐κB, nuclear factor κB; IL‐10, interleukin 10; IL‐10R, interleukin 10 receptor; TNF‐α, tumor necrosis factor‐α; M1, type 1 macrophages; M2, type 2 macrophages; antamiR‐466, antagomiR‐466 which specifically blocking miR‐466; antaCTL, A scramble antagomiR

We treated the mice with antagomiR‐466, which is specifically blocking miR‐466. We found that the influx of white cells (3.0 ± 0.2, Figure 8A) and neutrophils (2.9 ± 0.2, Figure 8B) in BALF at 24 h in antagomiR‐466‐pretreated group was significantly higher as compared with anta‐Ctl groups (1.5 ± 0.2 and 1.3 ± 0.2, respectively). The BALF levels of TNF‐α (463.3 ± 35.2), IL‐6 (662.1 ± 74.9), and total protein concentration (2.22 ± 0.5) were significantly increased in the antagomiR‐466 pretreated group (Figure 8C–E).

The overall findings reveal that once transferred from Ad‐MSC EVs to macrophages, miR‐466 inhibited TIRAP expression leading to downregulate MyD88‐NF‐κB axis, and therefore, tilting the balance toward M2 polarization (Figure 8F).

## 4 DISCUSSION

The main findings of this work are summarized as follows: (1) Intratracheal administration of Ad‐MSC EVs reduced the mortality of mice and decreased the total bacterial load, inflammation, lung protein permeability, and histological severity in mice with MDR‐PA pneumonia in a dose‐dependent manner; (2) therapeutic effects of high dose of Ad‐MSC EVs were equivalent to either Ad‐MSCs or h‐MSCs; (3) GW4869 or CD44 neutralizing antibody abrogated the immunomodulatory effects of Ad‐MSC EVs on macrophages, suggesting a critical role played by both the release of EV from MSCs, and their CD44‐mediated uptake by macrophages; (4) miR‐466 overexpression enhanced therapeutic benefits of Ad‐MSC EVs on mice survival, lung inflammation, protein permeability, histological severity, and macrophage phenotype switch to an alternate state type 2 profile; ( 5) when cocultured with h‐MSCs, CD14‐positive human monocytes derived from peripheral blood of patients with MDR‐PA‐induced pneumonia were more likely to differentiate toward type 2 macrophages than those derived from healthy people; (6) inhibition of the TIRAP‐MyD88‐NFκB axis induced by miR‐466 might account at least in part for the underlying mechanism of macrophage phenotypic switch.

This is the first study demonstrating that Ad‐MSC EVs are as effective as Ad‐MSCs in reducing inflammatory responses in a model of MDR‐PA pneumonia. Moreover, our results showed that miR‐466‐overexpressing MSC EVs reduced mortality in mice with severe MDR‐PA‐induced pneumonia. We previously reported that MSC EVs reduced the severity of endotoxin‐induced ALI in mice.^22^ However, the model was not lethal and both MSC EVs and endotoxin were given IT simultaneously.^30^ In order to translate a preclinical model closer to the real clinical situation, based on our first step of using a live bacteria‐induced severe pneumonia model,^20^ we used clinical MDR‐PA strain (O11 serotype) collected from the sputum specimen of a female pneumonia patient, instead of standard PA strain of PAO1.^15^ We also demonstrated beneficial effects of MSC EVs in a therapeutic model in which MSC EVs was administered as a therapy, 4 h following the MDR‐PA inoculation. The findings supported that intratracheal Ad‐MSC EVs given 4 h after bacterial inoculation were as effective as Ad‐MSCs in attenuating lung injury. Origin of MSCs (tissue or species) did not impact their beneficial effects. Our results open new perspectives in terms of developing innovative adjuvants alongside standard antibioterapy, particularly in the case of severe pneumonia induced by MDR‐PA.

To our knowledge, we provide the first evidence revealing that microRNAs mediated the therapeutic effects of Ad‐MSC EVs in a mouse model of ALI induced by MDR‐PA. Our microRNA expression profiling revealed that miR‐466 was the most highly expressed within Ad‐MSC EVs. Previous reports showed that miR‐466, first identified in embryonic stem cells, could upregulate IL‐10 expression by blocking RNA‐binding protein tristetraprolin, which inactivated *IL‐10* mRNA.^31^, ^32^, ^33^ MiR‐466 might therefore have the potential benefits to target GM‐CSF‐mediated inflammation by increasing the production of IL‐10 for promoting resolution of inflammation.^34^ Here in our study, in order to determine the contribution of miR‐466 delivery by EVs, we first pretreated Ad‐MSCs with miR‐466 mimics, thus increasing miR‐466 expression within the EVs. Administration of EVs from those pretreated Ad‐MSCs significantly enhanced the therapeutic effect of EVs, which was reversed by either secretion inhibitor of EVs (GW4869) or blocker of cell‐surface intake receptor (anti‐CD44 neutralizing antibody). The results indicated that the transfer of miR‐466 by EVs played a critical role in mediating the therapeutic effects of Ad‐MSCs in this mouse model of ALI induced by MDR‐PA. This result also emphasizes the crucial role played by small noncoding RNAs, such as microRNAs, in mediating the therapeutic effects of MSC‐EVs alongside other bioactive molecules contained in microvesicles, such as proteins, hormones, or mRNAs. We understand that the therapeutic mechanisms of EVs would be complex and interactive with each other in vivo. Although we found similar effects among some of indices between nature and transfected EVs (i.e., TNF‐alpha, IL‐10, and bacteria count), it was still possible to be a significant difference in overall survival rate. It would be interesting to further investigate any crosstalk pathways among various components within EVs.

To further investigate the mechanistic basis of this therapeutic effect, we were able to demonstrate that the miR‐466 microRNA contained in MSC‐EVs acts in particular by affecting the balance of the M1/M2 macrophage phenotype. In response to different signals, macrophages are subjected to a reprogramming and undergo two different polarization states that mirror the T helpers (Th) 1/Th2 nomenclature.^35^ It has been previously reported that in animal models of sepsis^36^, ^37^, ^38^ and pneumonia,^13^, ^14^, ^19^ MSCs have been shown to repolarize macrophages from M1 to M2 phenotype, which is characterized by high IL‐10, low TNF‐α expression, and increased capacity of phagocytosis. Similar to previous studies,^10^, ^20^, ^39^ this work demonstrated the capacity of Ad‐MSC EVs of skewing the macrophage phenotype toward a more immunomodulatory M2 state. However, this is the first time that the switch has been observed in a mouse model of ALI induced by MDR‐PA. Since Ad‐MSC EVs highly expressed miR‐466, we speculated that the transfer of miR‐466 from Ad‐MSC EVs to macrophage might play an important role in switching the macrophages phenotype. The fact that macrophages transfection with miR‐466 led to downregulation of M1 markers *iNOS* and *IL‐12*, upregulation of M2 markers *Arg1* and *IL‐10*, and a remarkable increase in the percentage of CD206 positive cells, corroborated our hypothesis. In addition, to go a step further, by luciferase assay, we observed that overexpression of miR‐466 could reduce activity of the vector which contained *TIRAP* and *MyD88* mRNA 3′UTR in transfected macrophages, demonstrating that miR‐466 might be the target toward *TIRAP* and *MyD88* mRNA. TIRAP is recruited to the plasma membrane by its phosphatidylinositol‐4,5‐bisphosphate binding domain, where it mediates recruitment of MyD88 to initiate Toll‐like receptors signaling.^40^, ^41^, ^42^, ^43^, ^44^ Regarding the downstream of MyD88/TIRAP pathways, we subsequently investigated two structural classes of NF‐κB protein expression, which played a key role in regulating the immune response to bacterial infection. Interestingly, miR‐466 significantly inhibited the expression of p65, one class of subunit with a transactivation function, and increased the expression of p50 and p105, the other class of subunit containing a number of ankyrin and having transrepression activity.^45^, ^46^ Although we can speculate that the differential effect of miR‐466 on the expression of the p50, p105, and p65 subunits can be explained by a particularly strong positive feedback on p105 and p50, this remains to be investigated. Overall, our findings confirmed our hypothesis, for the first time, that *TIRAP* was a functional downstream target of miR‐466, leading to downregulating of the NF‐κB signaling pathway, and as a result ultimately impacting macrophages polarization.

The current study has some limitations: (1) Scaling up the production process of MSC EVs to obtain sufficient quantity usable in the clinic remains challenging. Since different methods of isolation can influence vesicle yield, purity and contents, a consensus must be reached on the optimal culture conditions and EVs isolation protocols to standardize this process; (2) although some homologies exist between mice and humans cellular surface markers, both Arg‐1 and iNOS induction are confined mainly to murine macrophages^47^; (3) since we did not compare therapeutic benefits of EVs with antibiotic therapy or test EVs as an adjuvant therapy (in addition to antibiotics), whether MSC EVs are more potent, or add some synergy or additive effects with antibiotics remains unclear; (4) due to the small size of animals and the difficult vascular access,  the different treatments were delivered by intratracheal administration, a condition far from clinical conditions. Before going to humans, it is important to verify in big animals that the intravenous administration of EVs, EVs miR‐466, and MSCs have similar effects as the intratracheal administration; and (5) the difference in age between the patients and volunteers in the clinical part of our work is high. Unfortunately, because of the small sample size we did not have the possibility to make any adjustment between groups, it might impact the interpretation of the data, and might have introduced a bias in the results.

In conclusion, both Ad‐MSCs and Ad‐MSC EVs significantly reduced mortality rate of MDR‐PA pneumonia. Ad‐MSC‐derived EVs contain high level of miR‐466, which promote phenotype switch to M2 type of macrophages, exerting immunomodulatory effects and enhanced phagocytosis. Once transferred from Ad‐MSC EVs to macrophages, miR‐466 inhibited TIRAP expression leading to downregulate MyD88‐NF‐κB axis, and therefore, tilting the balance toward M2 polarization. Administration of Ad‐MSC EVs derived from Ad‐MSCs transfected with miR‐466 mimics further reduced mortality of MDR‐PA pneumonia. Such results may open a new therapeutic area for treating multidrug resistant hospital‐acquired and ventilator‐associated pneumonia.

## AUTHOR CONTRIBUTIONS

Meng‐meng Shi and Ying‐gang Zhu: Conception and design, collection and/or assembly of data, data analysis and interpretation, and manuscript writing; Antoine Monsel and Hanssa Summah: Conception and design, data analysis and interpretation, and manuscript writing; Jean‐Jacques Rouby and Jie‐ming Qu: Conception and design, financial support, data analysis and interpretation, manuscript writing, and final approval of manuscript.

## CONFLICT OF INTEREST

The authors declare no conflict of interest.

## Supporting information

Supporting Information

Supporting Information

Supporting Information

Supporting Information

Supporting Information

Supporting Information

## Data Availability

The data that support the findings of this study are available from the corresponding author upon reasonable request.
