# Supplementary material for: Role of miR‐466 in mesenchymal stromal cell derived extracellular vesicles treating inoculation pneumonia caused by multidrug‐resistant Pseudomonas aeruginosa
Source: Clin Transl Med. 2021 Jan 13;11(1):e287. doi: 10.1002/ctm2.287 (PMC7805403; doi:10.1002/ctm2.287)
Supplement: Supplementary file 1 — Supporting Information [file CTM2-11-e287-s001.docx]

**Supplementary methods**

***Collection of Human Peripheral Blood Monocytes.***

Monocytes were isolated from human peripheral blood by using magnetic bead separation methods according to manufacturers’ protocol. Briefly, peripheral blood mononuclear cells were collected from blood of MDR-PA infected patients and healthy donors by density gradient separation using Percoll (GE Healthcare Bio-sciences, Piscataway, NJ, USA). Red blood cells were lysed by incubating cells in ACK lysis buffer for 3 mins and mononuclear cells were washed with PBS. To reduce platelet contamination, cell suspensions were centrifuged at 400 g for 15 mins and cell pellets were resuspended and incubated with anti-human CD14 microbeads (Miltenyi Biotech, Auburn, CA, USA) for 15 mins at 4°C. After washing to remove unbound antibody, cell separation was done using autoMACS Pro Separator (Miltenyi Biotech). Purified CD14+ monocytes were plated into 6-well cell culture plates at a concentration of 0.5-1×10^6^ per well in RMPI 1640 media supplemented with 10% human serum blood type AB (Mediatech, Herndon, VA, USA). Cells were cultured for 5 days, without adding any cytokines, to generate macrophages with one change of media every 2 days after initiation of cultures at 37°C with 5% CO_2_.

***Isolation of Mouse adipose-derived MSCs.***

Mouse adipose-derived MSCs (Ad-MSCs) were isolated from mouse adipose tissue in line with previously reported methods [15]. Adipose tissue was harvested from the inguinal area and digested by 0.1% type I collagenase (Sigma-Aldrich, St. Louis, MO, USA, http://www. sigmaaldrich.com) in a 37°C water bath for 50 minutes, 200 rpm. Digested adipose was then centrifuged and re-suspended in Dulbecco’s modified Eagle’s medium and Nutrient Mixture F-12 (DMEM/F12, Gibco, Thermo Fisher Scientific, Waltham, MA, USA, http://www.thermofisher. com) supplemented with 10% fetal bovine serum (FBS, Gibco, Thermo Fisher Scientific, Waltham, MA, USA, http://www.thermofisher.com) and 1% penicillin/streptomycin (PS, Gibco, Thermo Fisher Scientific, Waltham, MA, USA, http://www.thermofisher.com). The re-suspended cells were plated on 10 cm dish (Corning, MA, USA, http://www.corning.com/asean/en/products/life-sciences.html) and cultured at 37°C in a 5% CO_2_ incubator. Twenty-four hours after plating, Ad-MSCs attached to the culture surface. Culture media was changed every other day until the cells were 80-90% confluent. Ad-MSCs were used for the experiments at the 4th or 5th passage [15]. Human bone marrow-derived MSCs (h-MSCs) were purchased from Sciencell Research Laboratories (Catalog #7500, <https://www.sciencellonline.com/human-bone-marrow-derived-mesenchymal-stem-cells.html>).

***Isolation of murine peritoneal macrophages.***

Peritoneal macrophages (primary PMs) were isolated from C57BL/6 male mice that were intraperitoneally injected with sterile sodium thioglycollate. Four days later, 10 ml phosphate buffered saline (PBS) was intraperitoneally injected to the mice and was then collected and centrifuged at 350 g for 5 min. The re-suspended cells were plated on 10 cm dish or 12-well cell culture plate (Corning, MA, USA, http://www.corning.com/asean/en/products/life-sciences.html) and cultured at 37°C in a 5% CO_2_ incubator. The adherent cells were used as macrophages.

***Strain Identification and Preparation.***

The clinical MDR-PA strain was incubated overnight in Lutia-Bertani (LB) broth, shaking at 37°C at the rate of 275rpm. 100μL inoculum was pipetted into 5ml fresh LB broth, shaking at 37°C at the same rate, for approximately 2 hours, with additional adjustment (either re-shaking or diluting) as needed, until optical density (OD) value (600nm) reached 0.8. Number of colony forming units (CFU) had previously been calculated: OD_600nm_=0.8 corresponds to 5×10^8^ CFU/ml. To prepare for the different levels of bacterial density, the adjusted inocula were centrifuged at 10,000g for 2 mins and pellets were subsequently re-suspended in PBS with the final density required.

***Murine Model of Pneumonia Induced by Intratracheal Inoculation with Multidrug-resistant* *Pseudomonas aeruginosa*.**

Animals were first anesthetized with intraperitoneal sodium pentobarbital (40 mg/kg) and then inoculated intratracheally (IT) with MDR-PA strain. To determine the four-day mortality of the experimental model, different bacterial doses were IT inoculated in four groups of eight mice: 0 CFU (PBS), 10^5^ CFU, 10^6^ CFU, 5×10^6^ CFU, 10^7^ CFU (final volume of 40 μL) using the protocol described previously [15]. In addition, 10^5^ and 10^6^ CFU were inoculated in four groups of five mice to determine the effect of increasing inoculum on BALF white blood cells, neutrophils, TNF-α, MIP-2, protein and bacterial count.

Four hours after MDR-PA IT inoculation, the mice were instilled intratracheally with 40 μL of either PBS, 8×10^5^ of h-MSCs, 8×10^5^ of Ad-MSCs, 2×Ad-MSC EVs, or L-929 EVs as a negative control. To determine optimal doses, an initial dose-response effect of IT Ad-MSCs and Ad-MSC EVs on MDR-PA inoculation pneumonia was performed. We also used Ad-MSCs and h-MSCs to confirm the different treatment effect. In separate experiments, IT 2×miR-466-m-MSC EVs or NS-m-MSC EVs (final volume of 40 μL) were given as a negative control.

***Measurement of Messenger RNA Expression.***

Total RNA for exploring messenger RNA (mRNA) expression was extracted using the TRIzol reagent (Invitrogen, Thermo Fisher Scientific, Waltham, MA, USA, http://www.thermofisher.com) according to the manufacturer’s instructions. RNA was eluted in RNase-free water and stored at -80°C. The expression of mRNA was detected by QuantiTect Reverse Transcription Kit and QuantiNova SYBR Green PCR Kit (Qiagen, Valencia, CA, https://www.qiagen.com). The separate well 2^-ΔΔCt^ cycle threshold method was used to determine relative quantitative levels of individual mRNA, and these were expressed as the fold difference to the *GAPDH*. Details about the sequences of *GAPDH*, *Arg-1*, *TNF-α*, *IL-12β*, *IL-10* and *iNOS* were described in **Supplementary Table 2.**

***Flow Cytometry Analysis.***

MDR-PA-primed human peripheral blood monocytes or murine macrophages co-incubated with either PBS, Ad-MSCs (2.5×105 cells) or transfected with miR-466 mimics (10μM) were collected respectively, washed with cold DPBS once, stained with APC-Cy7-conjugated Fixable Viability Stain 780 (BD HorizonTM) and incubated for 15 minutes at room temperature to assess cell viability. Cells were subsequently centrifuged twice at 800 g, 4°C for 5 minutes, stained with surface markers such as BV510-CD14 (Clone: MφP9), PE-Cy7-CD68 (Clone: Y1/82A), PE-CD86 (Clone: 2331 (FUN-1)), APC-CD206 (Clone:19.2, BD Pharmigen, San Diego, CA, <https://www.bdbiosciences.com>), BV421-F4/80 (Clone: T45-2342), PE-Cy7-CD11b (Clone: M1/70), PE-CD16/32 (Clone: 2.4G2), APC-CD206 (Clone: C068C2, BioLegend, San Diego, CA, https://www.biolegend.com), APC Rat IgG2a, κ Isotype Ctrl Antibody (APC-ISOtype) (Clone: RTK2758). For analysis of intracellular markers, such as PE-Cy7-CD68 (Clone: Y1/82A), PE-CD86 (Clone: 2331 (FUN-1)), APC-CD206 (Clone:19.2, BD Pharmigen, San Diego, CA, <https://www.bdbiosciences.com>), APC-CD206 (Clone: C068C2, BioLegend, San Diego, CA, https://www.biolegend.com), APC Rat IgG2a, κ Isotype Ctrl Antibody (APC-ISOtype) (Clone: RTK2758), cells were fixed with fixation buffer, permeabilized using permeabilization buffer and stained according to the manufacturer’s instructions. All the samples were acquired by CytExpert and analyzed by using flowjo Software.

***Western Blot Analysis.***

Primary antibodies used were MyD88 (D80F5) Rabbit mAb, NF-κB p65 (L8F6) Mouse mAb, NF-κB1 p105/p50 (D7H5M) Rabbit mAb, TIRAP (D6M9Z) Rabbit mAb (Mouse Specific) (<https://www.cst-c.com.cn>) and were purchased from Cell Signaling Technology (Cambridge, MA, USA, https://www.cst-c.com). Anti-alpha Tubulin antibody (<https://www.abcam.cn/alpha-tubulin-antibody-dm1a-loading-control-ab7291.html>) were purchased from Abcam (Cambridge, UK, https://www.abcam.com) as the control antibody. The Exosome identification kit for Western Blot were purchased from *Yeasen* Biotech Co., Ltd (Shanghai, CHINA, https://www.yeasen.com).We used 20ul of cell lysate and 20ug of proteins loaded for WB. The samples derived from cells were lysed in RIPA buffer, separated by electrophoresis on 10% SDS-PAGE gels and transferred to nitrocellulose (GE Amersham Biosciences, Piscataway, NJ). Proteins were detected by western blotting using primary antibodies at a concentration of 1/1000 (Cell Signaling Technology) or 1/10000 (abcam) and were incubated overnight at 4°C. Labeling of the first antibodies was detected using relevant secondary antibodies conjugated to HRP (Anti-rabbit IgG, HRP-linked antibody) (https://www.cst-c.com.cn/products/secondary-antibodies/7074) or anti-mouse IgG, HRP-linked antibody (https://www.cst-c.com.cn/products/secondary-antibodies/7076) according to the antibody sources and detected using ECL reagents (Thermo Fisher Scientific, Waltham, MA, USA, <https://www.thermofisher.com/order/catalog/product/32106>).

**Supplementary Figure Legends**

**Figure S1.** (A) Detailed antimicrobial resistance profile of MDR-PA strain. (B) Growth curve of MDR-PA strain. (C) Number of colony forming units (CFU) was calculated: OD_600nm_ = 1.0 corresponds to 8×10^8^ CFU/ml. (Left plate sample, 10^5^ times of diluted stock solution; Middle plate sample, 10^6^ times of diluted stock solution; Right plate sample, 10^7^ times of diluted stock solution).

PA: *pseudomonas aeruginosa*; OD: optical density.

**Figure S2.** (A-D) Cells were transfected with miR-466-m for 24 hours, followed by infection with MDR-PA for 6 hours. The mRNA expressions coding for M1 markers were analyzed by RT-PCR (A, N=4, *** indicates *p* < 0.001, *p* = 0.0005, 107.5 ± 7.1 for PA vs. 48.7 ± 3.6 for miR-466-m in RAW264.7, * indicates *p* < 0.05, *p* = 0.01, 67.6 ± 3.7 for PA vs. 45.5 ± 3.7 for miR-466-m in PM) (B, N=3, * indicates *p* < 0.05, *p* = 0.01, 19.8 ± 2.4 for PA vs. 8.2 ± 1.3 for miR-466-m in RAW264.7, * indicates *p* < 0.05, *p* = 0.04, 24.0 ± 2.0 for PA vs. 16.3 ± 1.4 for miR-466-m in PM). The mRNA expressions coding for M2 markers were analyzed by RT-PCR (C, N=4, ** indicates *p* < 0.01, *p* = 0.004, 2.0 ± 0.2 for PA vs. 9.1 ± 1.5 for miR-466-m in RAW264.7, ** indicates *p* < 0.01, *p* = 0.004, 5.1 ± 0.8 for PA vs. 12.2 ± 1.0 for miR-466-m in PM) (D, N=3, ** indicates *p* < 0.01, *p* = 0.002, 3.0 ± 0.7 for PA vs. 9.1 ± 0.5 for miR-466-m in RAW264.7, * indicates *p* < 0.05, *p* = 0.03, 20.5 ± 2.3 for PA vs. 32.6 ± 2.7 for miR-466-m in PM).

**Figure S3.** Dose response effect of Ad-MSCs on lung injury indices at 4 hours post inoculation. Data were represented as Mean ± SEM. (A) BAL white blood cells (*p* < 0.001, *** indicates *p* < 0.001, 1.3 ± 0.1, N=5 for 8×10^5^ Ad-MSCs vs. 3.2 ± 0.2, N=8 for PBS) and neutrophils (*p* < 0.001, *** indicates *p* < 0.001, 1.2 ± 0.1, N=5 for 8×10^5^ Ad-MSCs vs. 3.0 ± 0.2, N=8 for PBS) were significantly reduced with the optimal dose of 8×10^5^ of Ad-MSCs. (B) Total protein concentration in BALF was significantly reduced when treated with 8×10^5^ of Ad-MSCs (*p* = 0.0396, * indicates *p* < 0.05, 0.8 ± 0.1 for IT instillation of 8×10^5^ Ad-MSCs vs. 1.6 ± 0.2 for PBS, N=4). (C) The levels of TNF-α and MIP-2 in BALF were significantly reduced when treated with 8×10^5^ Ad-MSCs (*p* = 0.0114 for TNF-α, * indicates *p* < 0.05, 44.4 ± 6.1, N=6 for 8×10^5^ Ad-MSCs vs. 100.1 ± 21.1, N=5 for PBS; *p* <0.001 for MIP-2, *** indicates *p* < 0.001, 23.03 ± 3.5, N=9 for 8×10^5^ Ad-MSCs vs. 107.0 ± 17.4, N=4 for PBS) while the level of IL-10 in BALF were significantly increased (*p* = 0.0393, * indicates *p* < 0.05, 11.3 ± 1.1 for 8×10^5^ Ad-MSCs vs. 8.6 ± 0.2 for PBS, N=7).

BALF: bronchoalveolar lavage fluid; WBC: white blood cell; TNF-α: tumor necrosis factor-α; MIP-2: macrophage inflammatory protein 2; IL-10: interleukin 10; PA: *pseudomonas aeruginosa*; Ad-MSC: mouse adipose-derived mesenchymal stromal cells.

**Figure S4.** Dose response effect of Ad-MSC EVs on lung injury indices at 4 hours post inoculation. Data were represented as Mean ± SEM. (A) IT instillation of 2XAd-MSC EVs significantly reduced the influx of BAL white blood cells (*p* < 0.001, *** indicates *p* < 0.001, 1.2 ± 0.3, N=4 for 2XAd-MSC EVs vs. 2.9 ± 0.2, N=8 for PBS) as well as neutrophils (*p* <0.001, *** indicates *p* < 0.001, 1.1 ± 0.3, N=4 for 2XAd-MSC EVs vs. 2.9 ± 0.2, N=8 for PBS). (B) IT instillation of 2XAd-MSC EVs significantly reduced the levels of BAL TNF-α and MIP-2 (*p* < 0.001 for TNF-α, *** indicates *p* < 0.001, 45.4 ± 5.2, N=4 for 2XAd-MSC EVs vs. 144.3 ± 14.5, N=7 for PBS; *p* = 0.0409 for MIP-2, * indicates *p* < 0.05, 9.0 ± 2.2, N=5 for 2XAd-MSC EVs vs. 26.1 ± 6.2, N=6 for PBS) and increased the levels of BAL IL-10 (*p* = 0.0085, ** indicates *p* < 0.01, 9.8 ± 0.4, N=6 for 2XAd-MSC EVs s vs. 7.8 ± 0.5, N=7 for PBS).

BALF: bronchoalveolar lavage fluid; WBC: white blood cell; TNF-α: tumor necrosis factor-α; MIP-2: macrophage inflammatory protein 2; IL-10: interleukin 10; PA: *pseudomonas aeruginosa*; Ad-MSC EV: mouse adipose-derived mesenchymal stromal cells derived extracellular vesicles.

**Figure S5.** Effect of MSCs or EVs derived from different sources on lung injury indices at 4 hours post inoculation. Data were represented as Mean ± SEM. (A) Either Ad-MSCs or h-MSCs instillation significantly reduced the influx of BAL white blood cells (*p* = 0.0015, ** indicates *p* < 0.01, 1.3 ± 0.2, N=4 for Ad-MSCs vs. 2.7 ± 0.2, N=6 for PBS; *p* = 0.0089, ** indicates *p* < 0.01, 1.5 ± 0.2, N=5 for h-MSCs vs. PBS) and neutrophils (*p* < 0.001, *** indicates *p* < 0.001, 1.2 ± 0.1, N=5 for Ad-MSCs vs. 2.6 ± 0.2, N=6 for PBS; *p* = 0.0076, *** indicates *p* < 0.01, 1.4 ± 0.2, N=4 for h-MSCs vs. PBS), the levels of TNF-α (*p* = 0.0288, * indicates *p* < 0.05, 50.3 ± 10.9, N=7 for Ad-MSCs vs. 256.9 ± 126.7, N=3 for PBS; *p* = 0.365, NS indicates no significant, 146.4 ± 27.7, N=4 for h-MSCs vs. PBS) and MIP-2 (*p* = 0.005, ** indicates *p* < 0.01, 41.1 ± 11.8, N=6 for Ad-MSCs vs. 117.7 ± 11.5, N=3 for PBS; *p* = 0.0089, ** indicates *p* < 0.01, 41.2 ± 13.7, N=5 for h-MSCs vs. PBS). (B) Both Ad-MSC EVs and h-MSC EVs instillation significantly reduced the influx of BAL white blood cells (*p* = 0.0334, * indicates *p* < 0.05, 1.1 ± 0.3, N=4 for Ad-MSC EVs vs. 2.4 ± 0.4, N=7 for PBS; *p* = 0.0273, * indicates *p* < 0.05, 1.2 ± 0.2, N=5 for h-MSC EVs vs. PBS) and neutrophils (*p* = 0.0265, * indicates *p* < 0.05, 1.0 ± 0.2, N=4 for Ad-MSC EVs vs. 2.3 ± 0.4, N=7 for PBS; *p* =0.0231, * indicates *p* < 0.05, 1.1 ± 0.2, N=5 for h-MSC EVs vs. PBS), the levels of TNF-α (*p* = 0.0117, * indicates *p* < 0.05, 22.2 ± 11.6, N=4 for Ad-MSC EVs vs. 104.3 ± 15.9, N=4 for PBS; *p* = 0.005, ** indicates *p* < 0.01, 11.1 ± 7.5, N=4 for h-MSC EVs vs. PBS) and MIP-2 (*p* < 0.001, *** indicates *p* < 0.001, 9.0 ± 2.2, N=5 for Ad-MSC EVs vs. 117.7 ± 11.5, N=3 for PBS; *p* < 0.001, *** indicates *p* < 0.001, 13.0 ± 2.4, N=5 for h-MSC EVs vs. PBS).

BALF: bronchoalveolar lavage fluid; WBC: white blood cell; TNF-α: tumor necrosis factor-α; MIP-2: macrophage inflammatory protein 2; PA: pseudomonas aeruginosa; Ad-MSC: mouse adipose-derived mesenchymal stromal cells; h-MSC: human bone marrow-derived mesenchymal stromal cells.

**Figure S6.** Dose response effect of MDR-PA on lung injury indices at 24 or 36 hours post inoculation. Data were represented as Median ± SEM. (A) BALF white blood cells were significantly increased with higher dose of inoculum (*p* = 0.0021, ** indicates *p* < 0.01, 2.3 ± 0.1 for IT instillation of 10^6^ CFU vs. 0.5 ± 0.1 for 10^5^ CFU, N=5), as well as neutrophils (*p* = 0.0015, ** indicates *p* < 0.01, 2.0 ± 0.02 for IT instillation of 10^6^ CFU vs. 0.5 ± 0.1 for 10^5^ CFU, N=5). (B) Bacterial load in BALF were significantly increased with higher dose of inoculum (*p* = 0.0445, * indicates *p* < 0.05, 391,333 ± 48,667 for IT instillation of 10^6^ CFU vs. 106,750 ± 9,250 for 10^5^ CFU, N=5). (C) The levels of TNF-α and MIP-2 in BALF were significantly increased with higher dose of inoculum (*p* = 0.029 for TNF-α, * indicates *p* < 0.05, 91.1 ± 16.7 for IT instillation of 10^6^ CFU vs. 14.5 ± 1.5 for 10^5^ CFU, N=5; *p* = 0.009 for MIP-2, ** indicates *p* < 0.01, 16.3 ± 0.2 for IT instillation of 10^6^ CFU vs. 4.3 ± 1.1 for 10^5^ CFU, N=5). No statistically difference was found in the total protein concentration in BALF between groups (*p* = 0.1085, NS indicates no significant, 1.1± 0.2 for IT instillation of 10^6^ CFU vs. 0.5 ± 0.03 for 10^5^ CFU, N=5).

BALF: bronchoalveolar lavage fluid; WBC: white blood cell; TNF-α: tumor necrosis factor-α; MIP-2: macrophage inflammatory protein 2.

**Figure S7.** (A) Kaplan-Meier survival curves of MDR-PA infected C57BL/6 mice. Mice were IT instilled with different bacterial doses, 10^5^ CFU, 10^6^ CFU, 5×10^6^ CFU, 10^7^ CFU, respectively. Survival was determined up to 96 hours (*p* < 0.0001, N=8). (B) Data was presented as median value with interquartile range. IT instillation of Ad-MSCs or 2×Ad-MSC EVs 4 hours post-infection significantly reduced the influx of BAL white blood cells (*p* = 0.008, ** indicates *p* < 0.01, 1.3 [1.1-1.8] for IT instillation of Ad-MSCs vs. 2.8 [2.2-3.3] for PA; *p* = 0.008, ** indicates *p* < 0.01, 1.2 [0.7-1.5] for IT instillation of Ad-MSC EVs vs. 2.8 [2.2-3.3] for PA, N=5 for PA, N=5 for Ad-MSCs, N=5 for Ad-MSC EVs, N=5 for L-929 EVs) as well as neutrophils (*p* = 0.008, ** indicates *p* < 0.01, 1.2 [1.0-1.5] for IT instillation of Ad-MSCs vs. 2.7 [2.1-3.2] for PA; *p* = 0.008, ** indicates *p* < 0.01, 1.1 [0.6-1.6] for IT instillation of Ad-MSC EVs vs. 2.7 [2.1-3.2] for PA, N=5 for PA, N= 5 for Ad-MSCs, N=5 for Ad-MSC EVs, N=5 for L-929 EVs). (C) IT Ad-MSCs or Ad-MSC EVs decreased the BAL levels of TNF-α (*p* = 0.03, * indicates *p* < 0.05, 43.3 [20.5-69.3] for IT instillation of Ad-MSCs vs. 78.2 [54.0-124.2] for PA; *p* = 0.004, ** indicates *p* < 0.01, 10.1 [3.5-28.3] for IT instillation of Ad-MSC EVs vs. 78.2 [54.0-124.2] for PA, N=6 for PA, N=6 for Ad-MSCs, N=5 for Ad-MSC EVs, N=5 for L-929 EVs), as well as the levels of MIP-2 (*p* = 0.04, * indicates *p* < 0.05, 33.3 [12.2-37.8] for IT instillation of Ad-MSCs vs. 110.7 [102.2-140.2] for PA; *p* = 0.04, * indicates *p* < 0.05, 9.3 [7.9-13.0] for IT instillation of Ad-MSC EVs vs. 110.7 [102.2-140.2] for PA, N=3 for PA, N=5 for Ad-MSCs, N=5 for Ad-MSC EVs, N=5 for L-929 EVs). IT Ad-MSCs or Ad-MSC EVs significantly increased the BALF level of IL-10 (*p* = 0.02, * indicates *p* < 0.05, 6.0 [4.9-6.4] for IT instillation of Ad-MSCs vs. 1.6 [1.3-1.8] for PA; *p* = 0.008, ** indicates *p* < 0.01, 5.3 [4.2-6.9] for IT instillation of Ad-MSC EVs vs. 1.6 [1.3-1.8] for PA, N=5 for PA, N=5 for Ad-MSCs, N=5 for Ad-MSC EVs, N=5 for L-929 EVs) at 24 hours, respectively, as compared with PBS. (D) Total protein concentration was significantly decreased when instilled IT Ad-MSCs or Ad-MSC EVs (*p* = 0.009, ** indicates *p* < 0.01, 0.7 [0.7-1.0] for IT instillation of Ad-MSCs vs. 1.6 [1.2-1.9] for PA; *p* = 0.002, ** indicates *p* < 0.01, 0.5 [0.4-0.6] for IT instillation of Ad-MSC EVs vs. 1.6 [1.2-1.9] for PA, N=6 for PA, N=5 for Ad-MSCs, N=5 for Ad-MSC EVs, N=5 for L-929 EVs). (E) Mice treated with Ad-MSCs or Ad-MSC EVs significantly decreased the bacterial load in BALF (*p* = 0.01, * indicates *p* < 0.05, 295,600 ± 29,334 for IT instillation of Ad-MSCs vs. 983,000 ± 226,853 for PA; *p* = 0.02, *indicates *p* < 0.05, 48,667 ± 28,603 for IT instillation of Ad-MSC EVs vs. 983,000 ± 226,853 for PA, N=4 for PA, N=5 for Ad-MSCs, N=4 for Ad-MSC EVs, N=4 for L-929 EVs). (F) Photographs depicting the bacterial colony forming units in petri-dish plates for the 4 experimental groups: mice instilled with PBS, Ad MSCs, Ad-MSC EVs or L-929 EVs, 4 hours following the MDR-PA instillation. (G) Lung injury score was significantly decreased (*p* < 0.0001, *** indicates *p* < 0.001, 0.252 ± 0.0337 for IT instillation of Ad-MSCs vs. 0.873 ± 0.0463 for PA; *p* <0.0001, *** indicates *p* < 0.001, 0.234 ± 0.0221 for IT instillation of Ad-MSC EVs vs. 0.873 ± 0.0463 for PA, N=6 for PA, N=5 for Ad-MSCs, N=5 for Ad-MSC EVs, N=6 for L-929 EVs). (H) When treated with Ad-MSCs or Ad-MSC EVs, the histology showed less inflammatory cells infiltrating inter-alveolar septa and respecting alveolar space and lung architecture.

BALF: bronchoalveolar lavage fluid; WBC: white blood cell; TNF-α: tumor necrosis factor-α; MIP-2: macrophage inflammatory protein 2; IL-10: interleukin 10; PA: *pseudomonas aeruginosa*; Ad-MSC: mouse adipose-derived mesenchymal stromal cells; Ad-MSC EV: mouse adipose-derived mesenchymal stromal cells derived extracellular vesicles.

**Figure S8.** (A) miR-466-m-MSC EVs treatment reduced blood white blood cells by 36% (B) miR-466-m-MSC EVs treatment reduced neutrophils by 40%, but the difference did not reach statistical significance (*p* = 0.2, 1.2 ± 0.2 for IT instillation of miR-466-m-MSC EVs vs. 1.9 ± 0.2 for NS-m-MSC EVs, N=5 for PA, N=5 for miR-466-m-MSC EVs, N=5 for NS-m-MSC EVs; *p* = 0.1, 1.0 ± 0.2 for miR-466-m-MSC EVs vs. 1.6 ± 0.2 for NS-m-MSC EVs, N=5 for PA, N=5 for miR-466-m-MSC EVs, N=5 for NS-m-MSC EVs).

**Supplementary Video**

The video loops showed three-dimensional fluorescent confocal imaging of intracellular bacteria within murine macrophages. DAPI (BLUE) was used to stain the nucleus. Anti-alpha Tubulin antibody (RED) was used to stain the cytoskeleton. GREEN represented GFP-PA.

**Supplementary Table 1**

Protocol of microRNA transfection by Lipofectamine™ RNAiMAX reagent

| Component | 24-well | 12-well | 6-well |
| --- | --- | --- | --- |
| Adherent cells | 0.5-2×10^5^ | 2-5×10^5^ | 0.25-1×10^6^ |
| Opti-MEM medium | 50μL | 100μL | 150μL |
| Lipofectamine™ RNAiMAX reagent | 3μL | 6μL | 9μL |
| Opti-MEM medium | 50μL | 100μL | 150μL |
| microRNA (10uM) | 1(10pmol) | 2(20pmol) | 3(30pmol) |
| Diluted microRNA | 50μL | 100μL | 150μL |
| Diluted Lipofectamine™ RNAiMAX reagent | 50μL | 100μL | 150μL |
|  | Incubation at room temperature for 5 mins | | |
| microRNA-lipid complex per well | 50μL | 100μL | 250μL |
| Final microRNA used per well | 5pmol | 10pmol | 25pmol |
| Final Lipofectamine™ RNAiMAX reagent used per well | 1.5μL | 3μL | 7.5μL |

**Supplementary Table 2**

Sequence of primers

| Gene |  | Sequence 5’-3’ |
| --- | --- | --- |
| *GAPDH* | F | TGTGAACGGATTTGGCCGTA |
|  | R | ACTGTGCCGTTGAATTTGCC |
| *Arg-1* | F | CAGAAGAATGGAAGAGTCAG |
|  | R | CAGATATGCAGGGAGTCACC |
| *MyD88* | F | TCGAGTTTGTGCAGGAGATG |
|  | R | AGGCTGAGTGCAAACTTGGT |
| *IL-12* | F | TGGTTTGCCATCGTTTTGCTG |
|  | R | ACAGGTGAGGTTCACTGTTTCT |
| *IL-10* | F | GCTGGACAACATACTGCTAACC |
|  | R | ATTTCCGATAAGGCTTGGCAA |
| *iNOS* | F | ACATCGACCCGTCCACAGTAT |
|  | R | CAGAGGGGTAGGCTTGTCTC |
| mmu-Tirap-3’UTR | F | CGGCTCGAGGGGAATAGCTCACAGCAGTCAT |
|  | R | AATGCGGCCGCCCTCGGGGTGCATTTTTAAT |
| mmu-Tirap-3’UTR-MUT | F | GTGTGTGAGTGTGTGTACTGAAACTCTTCCCCAGA |
|  | R | CACACACTCACACACAAGGACTGGATACTTGCCAC3 |

**Supplementary Table 3**

Demographic information of patients

| Patient no. | Sex | Age |
| --- | --- | --- |
| 1 | Male | 75 |
| 2 | Female | 69 |
| 3 | Female | 88 |
| 4 | Male | 68 |
| 5 | Male | 83 |
| 6 | Male | 66 |

Demographic information of volunteers

| Volunteers no. | Sex | Age |
| --- | --- | --- |
| 1 | Male | 25 |
| 2 | Female | 26 |
| 3 | Female | 34 |
| 4 | Male | 35 |
| 5 | Female | 25 |
| 6 | Male | 27 |
