# Supplementary material for: Role of miR‐466 in mesenchymal stromal cell derived extracellular vesicles treating inoculation pneumonia caused by multidrug‐resistant Pseudomonas aeruginosa
Source: Clin Transl Med. 2021 Jan 13;11(1):e287. doi: 10.1002/ctm2.287 (PMC7805403; doi:10.1002/ctm2.287)

Supplementary Figure S9

A

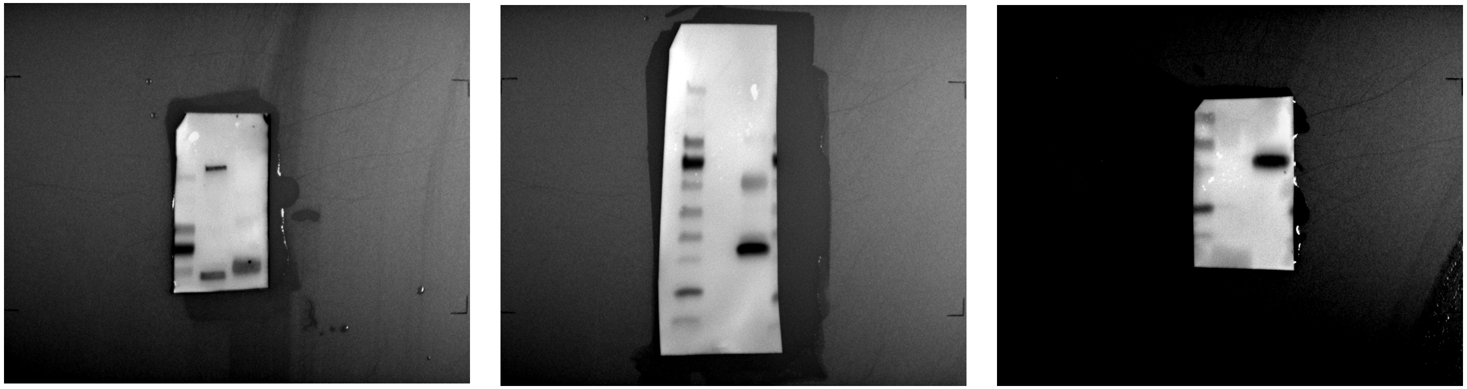

CD9 CD63 TSG101

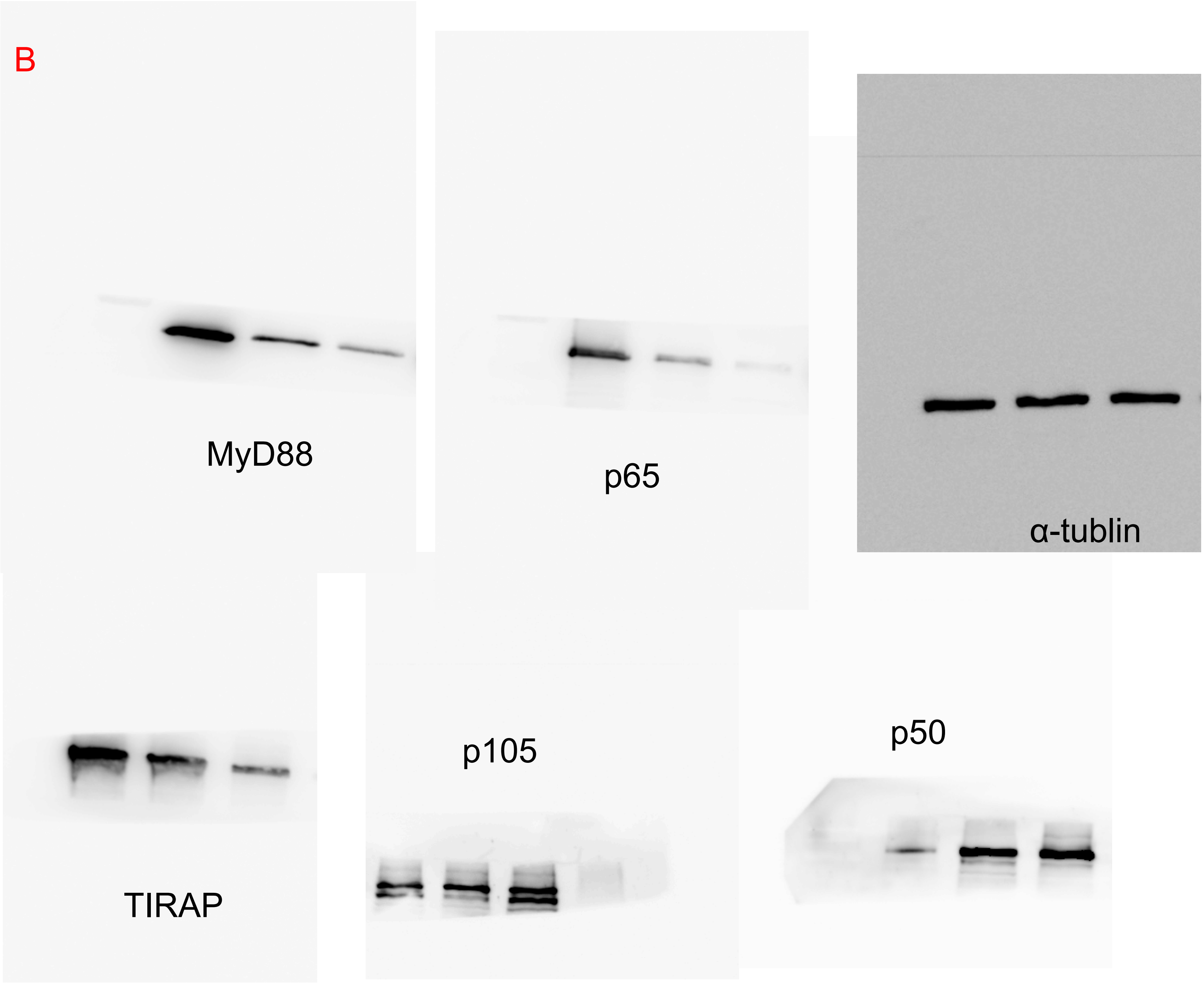
B
MyD88
p65
α-tublin
TIRAP
p105
p50

C

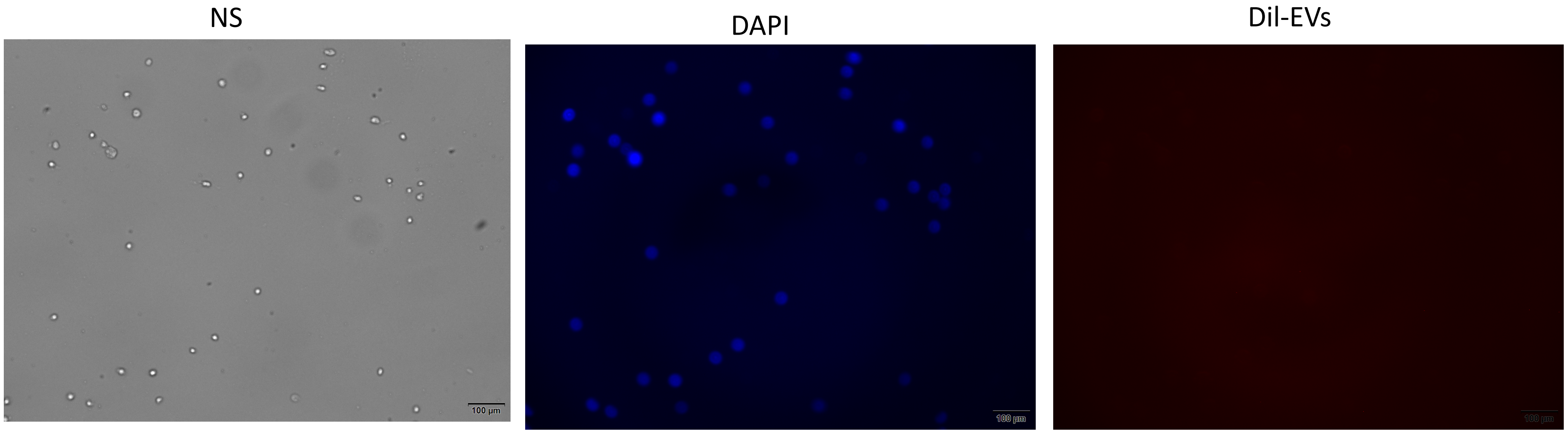

D

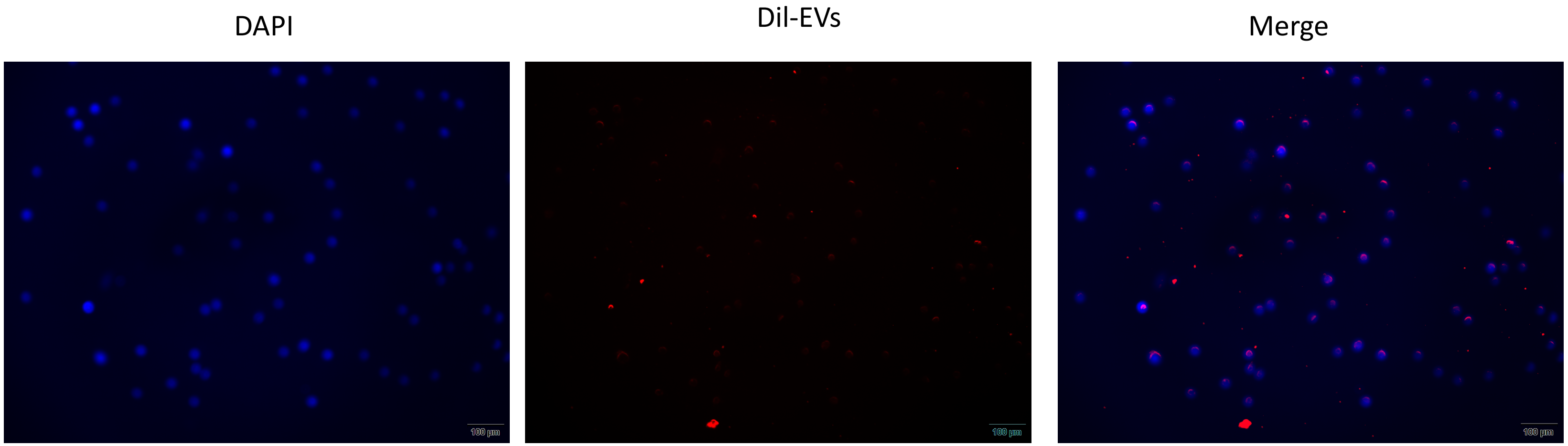

Supplement: Supplementary file 2 — Supporting Information [file CTM2-11-e287-s006.pdf]
